# Supplementary material for: Comparison of NSG-Quad and MISTRG-6 humanized mice for modeling circulating and tumor-infiltrating human myeloid cells
Source: Mol Ther Methods Clin Dev. 2025 May 14;33(2):101487. doi: 10.1016/j.omtm.2025.101487 (PMC12152875; doi:10.1016/j.omtm.2025.101487)
Supplement: Document S1. Figures S1–S9 [file mmc1.pdf]

OMTM, Volume *33*

Supplemental information

Comparison of NSG-Quad and MISTRG-6 humanized mice for modeling circulating and tumor-infiltrating human myeloid cells

Anna Chen, Viktoria Knöbl, Oliver Walzer, Jana Hauser, Ines Neuwirth, Magdalena Frank, Nina Braun, Semina Duvnjak, Johannes Reisecker, Carmen Stecher, Alex Farr, Christine Brostjan, and Dietmar Herndler-Brandstetter

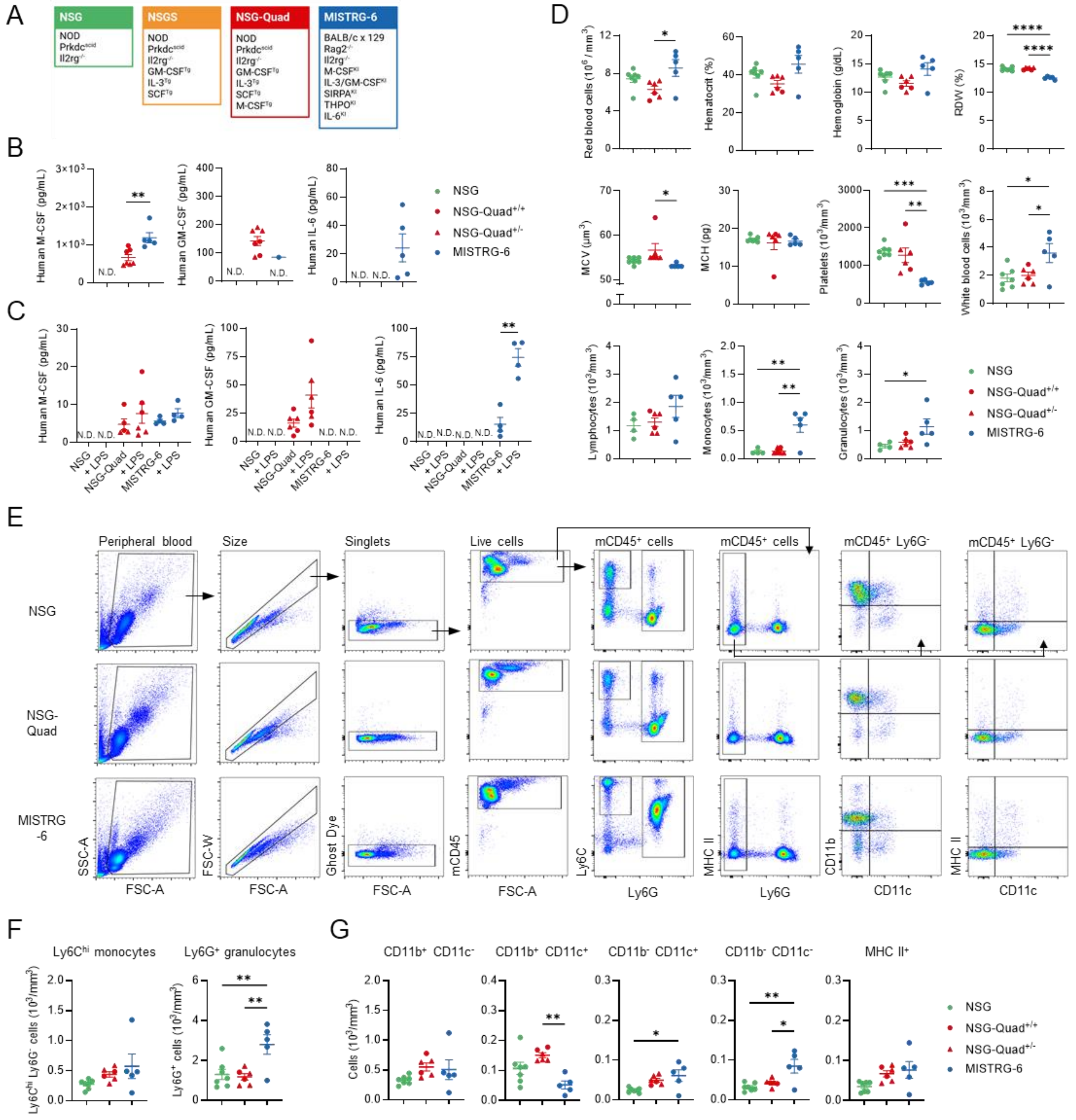

Figure S1: Characterization of NSG, NSG-Quad and MISTRG-6 mice (related to Figure 1). (**A**) Schematic illustration of the genetic background of NSG, NSGS (NSG-SGM3), NSG-Quad and MISTRG-6 mice. (**B**) Quantification of human M-CSF, GM-CSF and IL-6 protein in the plasma of 16-20 week old female NSG (n=5), NSG-Quad (dots, M-CSF$^{+/+}$ n=3; triangles, M-CSF$^{+/-}$ n=4) and MISTRG-6 mice (n=5). (**C**) Quantification of human M-CSF, GM-CSF and IL-6 protein in the supernatant of unstimulated and LPS-stimulated bone marrow cells from 16-20 week old female NSG (n=3), NSG-Quad (n=6) and MISTRG-6 mice (n=4). Triangle symbols

represent human M-CSF$^{+/-}$ mice. (**D**) Analysis of mouse red blood cells, hematocrit, hemoglobin, RDW, MCV, MCH, platelets, as well as mouse white blood cells, lymphocytes, monocytes and granulocytes in the blood of 16-20 week old female NSG (n=7), NSG-Quad (n=6) and MISTRG-6 mice (n=5). Triangle symbols represent human M-CSF$^{+/-}$ mice. (**E**) Representative flow cytometry plots showing the gating strategy for identifying mouse immune cell subsets in the blood of NSG, NSG-Quad and MISTRG-6 mice. (**F**) Absolute numbers of mouse Ly6C^{hi} Ly6G^{-} monocytes and Ly6G^{+} granulocytes in the blood of 16-20 week old female NSG (n=7), NSG-Quad (n=6) and MISTRG-6 mice (n=5). (**G**) Absolute numbers of mouse myeloid cell subsets in the blood of 16-20 week old female NSG (n=7), NSG-Quad (n=6) and MISTRG-6 mice (n=5). Data are shown as mean ± SEM. *P* values were calculated using one-way ANOVA with Tukey's multiple comparison test. * $P < 0.05$, ** $P < 0.01$, *** $P < 0.001$ and **** $P < 0.0001$. Abbreviations: CSF, colony-stimulating factor; GM-CSF, granulocyte-macrophage colony-stimulating factor; IL2rg, interleukin 2 receptor subunit gamma; KI, knock-in; MCH, mean corpuscular hemoglobin; M-CSF, macrophage colony-stimulating factor; MCV, mean corpuscular volume; N.D., not detected; NOD, non-obese diabetic; Prkdc, protein kinase DNA-activated catalytic subunit; Rag2, recombination activating gene 2; RDW, red blood cell distribution width; SCF, stem cell factor; scid, severe combined immunodeficiency; SIRPA, signal-regulatory protein alpha; Tg, transgene; THPO, thrombopoietin.

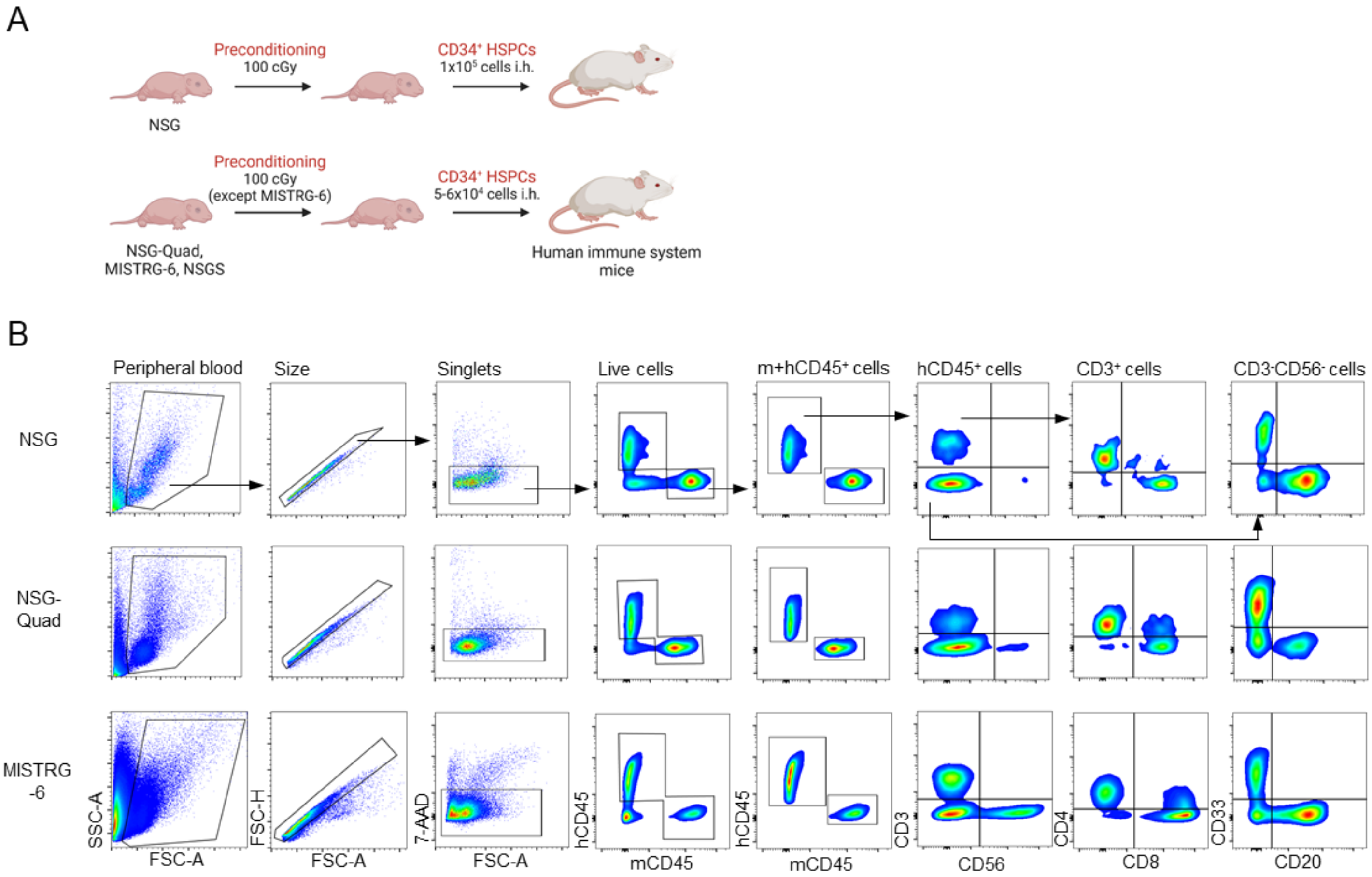

Figure S2: Human hematopoietic cell engraftment scheme and identification of human immune cell populations in NSG, NSG-Quad and MISTRG-6 mice (related to Figure 1). (**A**) Schematic illustration of the engraftment protocol with human cord blood-derived CD34^{+} HSPCs into NSG, NSGS, NSG-Quad and MISTRG-6 mice. (**B**) Representative flow cytometry plots showing the gating strategy for identifying human immune cell subsets in the blood of NSG, NSG-Quad and MISTRG-6 mice. Abbreviations: cGy, centi Gray; HSPC, hematopoietic stem and progenitor cells; i.h., intrahepatic.

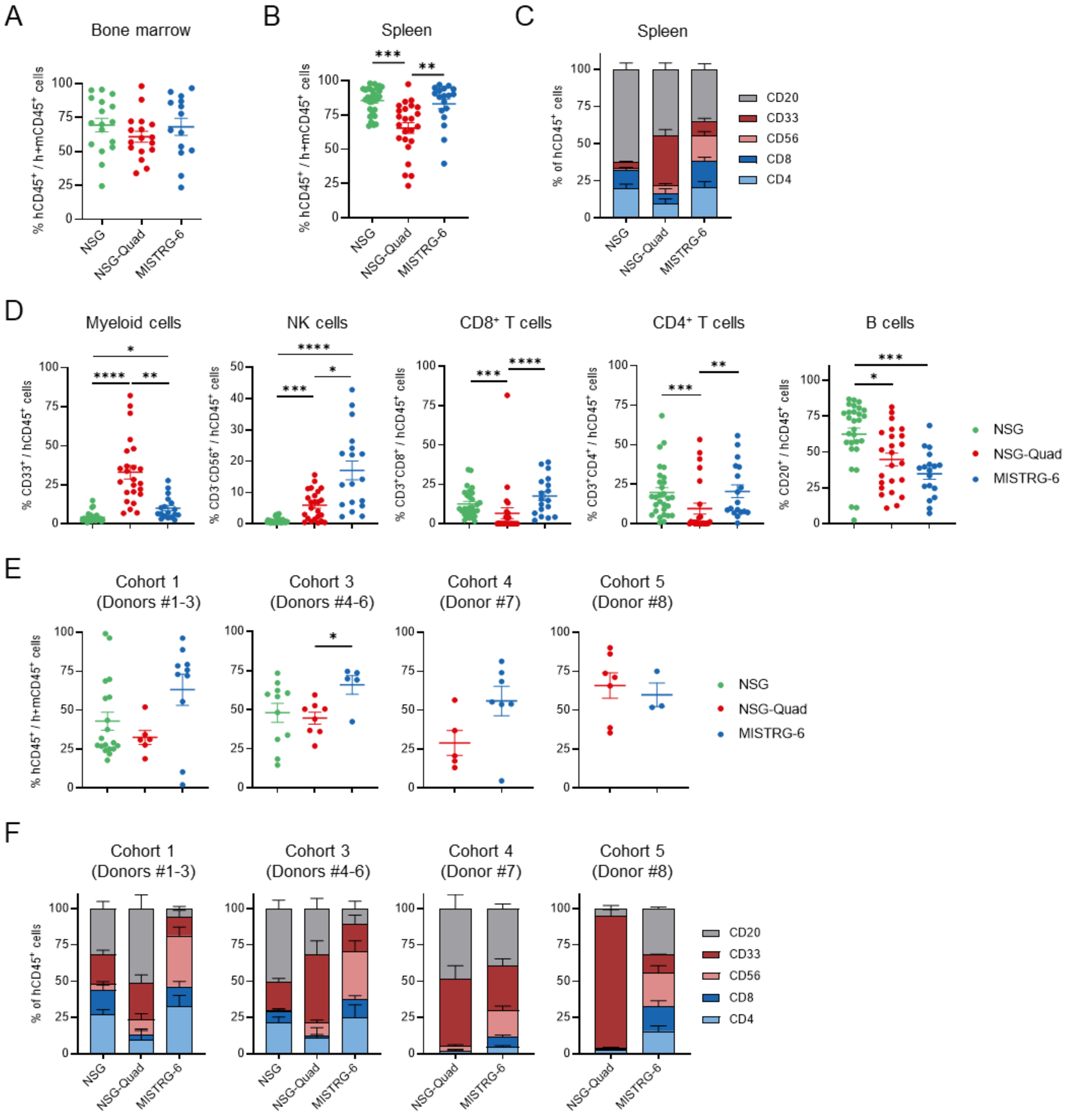

Figure S3: Human hematopoietic cell engraftment in the bone marrow, spleen and blood of NSG, NSG-Quad and MISTRG-6 mice (related to Figure 1). (**A**) Percentage of human CD45$^+$ (hCD45$^+$) cells of total CD45$^+$ cells (mouse and human) in the bone marrow of NSG (n=17), NSG-Quad (n=17) and MISTRG-6 mice (n=14) 10-15 weeks post engraftment with human cord blood-derived CD34$^+$ cells. (**B**) Percentage of human CD45$^+$ (hCD45$^+$) cells of total CD45$^+$ cells (mouse and human) in the spleen of NSG (n=29), NSG-Quad (n=24) and MISTRG-6 mice (n=18) 10-15 weeks post engraftment with human cord blood-derived CD34$^+$ cells. (**C**) Human immune cell composition in the spleen of NSG (n=29), NSG-Quad (n=24) and MISTRG-6 mice (n=18) 10-15 weeks post engraftment. (**D**) Percentage of human immune cell subsets in the spleen of

NSG, NSG-Quad and MISTRG-6 mice 10-15 weeks post engraftment (data from C). B cells ($CD3^-CD56^-CD33^-CD20^+$), myeloid cells ($CD3^-CD56^-CD20^-CD33^+$), NK cells ($CD3^-CD56^+$), $CD8^+$ T cells ($CD3^+CD4^-CD8^+$) and $CD4^+$ T cells ($CD3^+CD8^-CD4^+$) (data from C). (**E**) Percentage of human $CD45^+$ ($hCD45^+$) cells of total $CD45^+$ cells in the blood of NSG (n=11-19), NSG-Quad (n=5-8) and MISTRG-6 mice (n=3-10) 10-15 weeks post engraftment split by $CD34^+$ cell donors. (**F**) Human immune cell composition in the blood of NSG, NSG-Quad, and MISTRG-6 mice 10-15 weeks post engraftment split by $CD34^+$ cell donors (data from E). Data are shown as mean ± SEM. *P* values were calculated using Kruskal-Wallis with Dunn's correction test (A, B, D, E) and two-tailed, unpaired Student's *t*-test (E). * $P < 0.05$, ** $P < 0.01$, *** $P < 0.001$ and **** $P < 0.0001$.

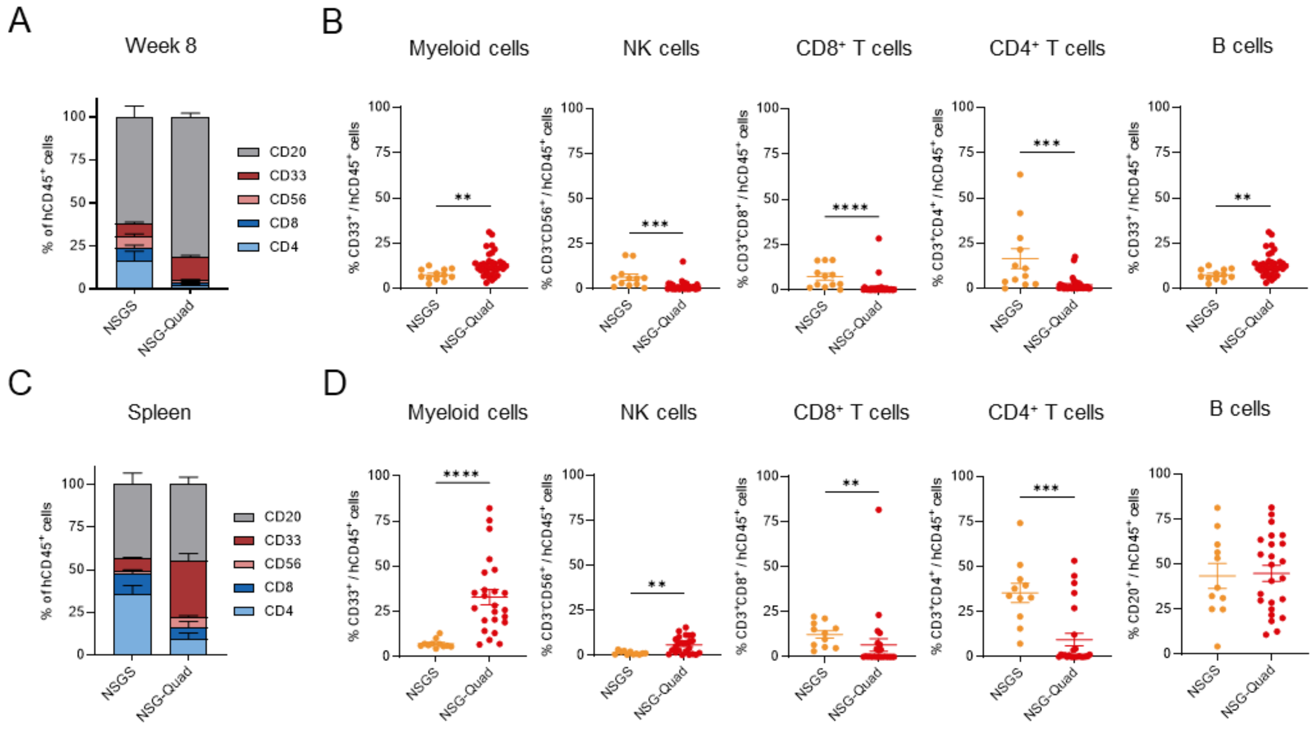

Figure S4: Human immune cell composition in NSGS and NSG-Quad humanized mice (related to Figure 2). (**A**) Human immune cell composition in the blood of NSGS (n=12) and NSG-Quad mice (n=34) 8 weeks post engraftment. (**B**) Percentage of human immune cell subsets in the blood of NSGS and NSG-Quad mice 8 weeks post engraftment (data from A). (**C**) Human immune cell composition in the spleen of NSGS (n=11) and NSG-Quad mice (n=24) 10-15 weeks post engraftment. (**D**) Percentage of human immune cell subsets in the spleen of NSGS and NSG-Quad mice 10-15 weeks post engraftment (data from C). Data are shown as mean ± SEM. *P* values were calculated using two-tailed, unpaired Mann-Whitney U test. ** $P < 0.01$, *** $P < 0.001$ and **** $P < 0.0001$.

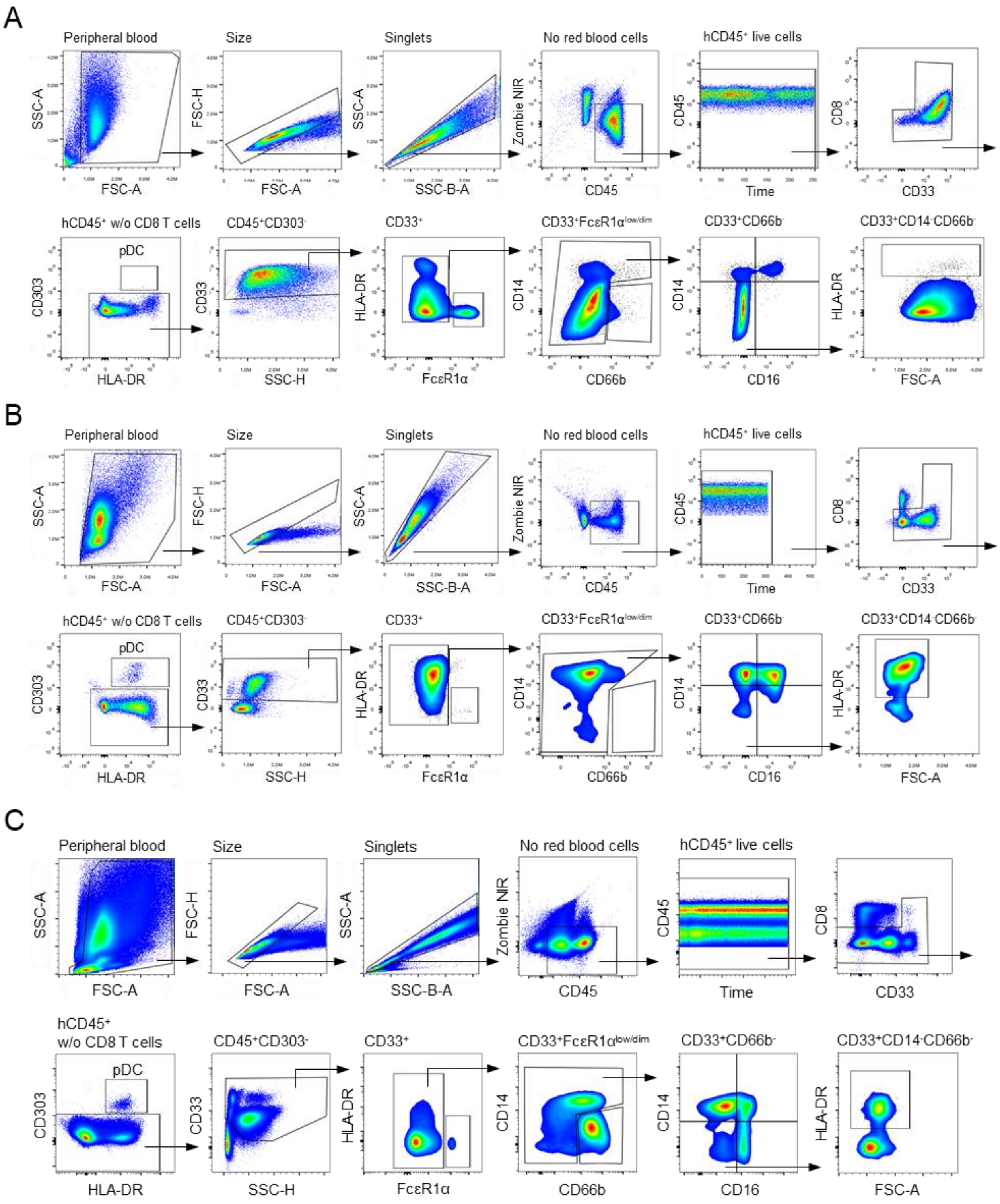

Figure S5: Gating strategy of human myeloid cell subpopulations in NSG-Quad, MISTRG-6 and healthy human adults (related to Figure 3). (**A**) Representative flow cytometry plots showing the gating strategy of human myeloid cell subpopulations in the blood of NSG-Quad mice. (**B**) Representative flow cytometry plots showing the gating strategy of human myeloid cell subpopulations in the blood of MISTRG-6 mice. (**C**) Representative flow cytometry plots showing the gating strategy of human myeloid cell subpopulations in the blood of healthy human adults.

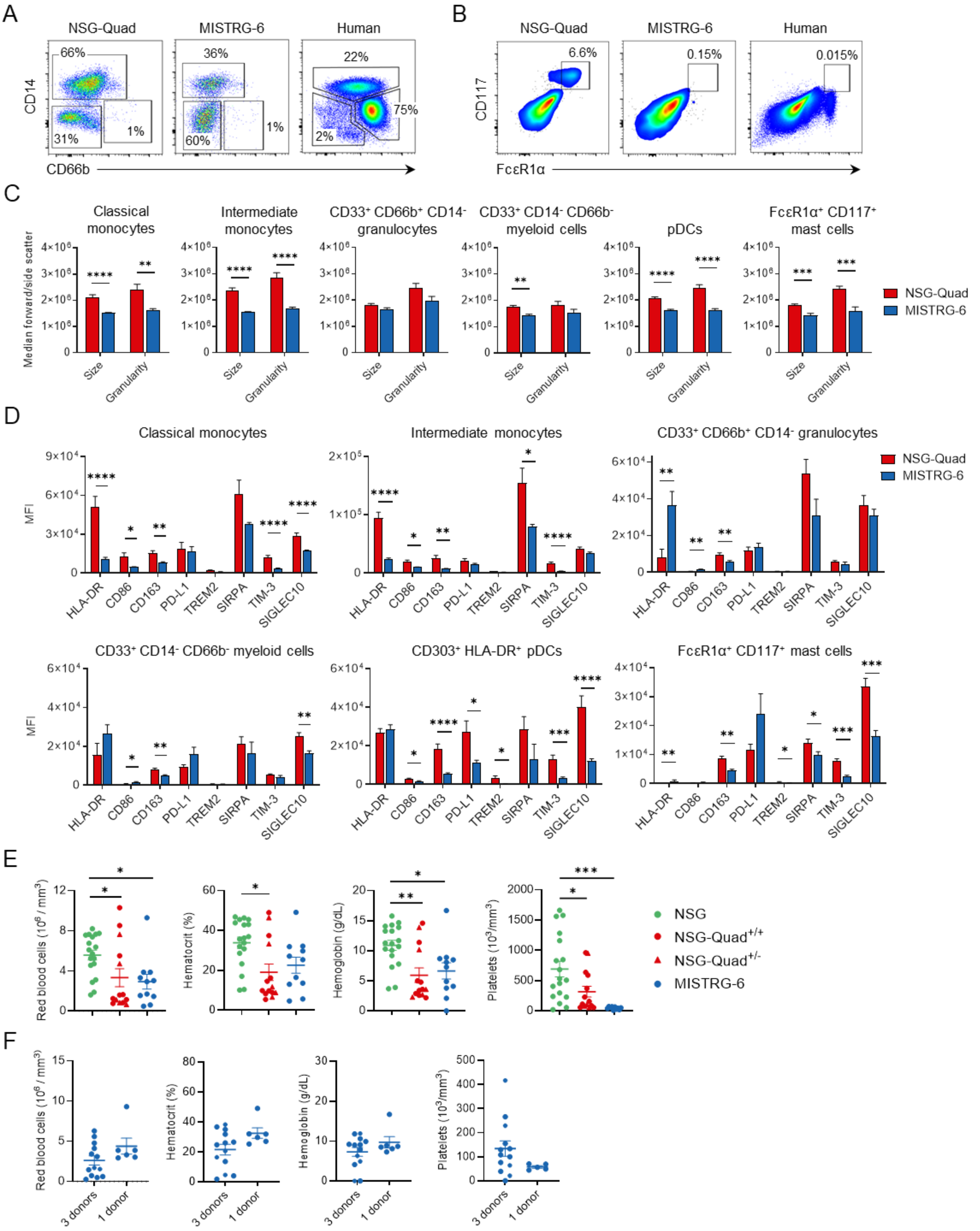

Figure S6: Expression pattern of key molecules in human myeloid cell subpopulations in NSG-Quad

and MISTRG-6 humanized mice (related to Figure 3). (**A**) Representative flow cytometry plots of $CD14^+$ monocytes, $CD66b^+$ granulocytes and $CD33^+CD14^-CD66b^-$ myeloid cells in the blood of NSG-Quad, MISTRG-6 and a healthy human adult. (**B**) Representative flow cytometry plots of $Fc\varepsilon R1\alpha^+CD117^+$ mast cells in NSG-Quad, MISTRG-6 and a healthy human adult. (**C**) Median forward scatter (cell size) and median side scatter (granularity) of six human myeloid cell populations in the blood of NSG-Quad (n=18) and MISTRG-6 mice (n=16) at 10-15 weeks post engraftment with human cord blood-derived $CD34^+$ cells. (**D**) Median fluorescence intensity (MFI) of eight key myeloid molecules on six human myeloid cell populations in the blood of NSG-Quad (n=18; mast cells n=15) and MISTRG-6 mice (n=16; mast cells n=12) at 10-15 weeks post engraftment with human cord blood-derived $CD34^+$ cells. (**E**) Analysis of mouse red blood cells, hematocrit, hemoglobin and platelets in the blood of hCD34-engrafted NSG (n=18), NSG-Quad (n=14) and MISTRG-6 mice (n=11). Triangle symbols represent M-$CSF^{+/-}$ mice. (**F**) Analysis of mouse red blood cells, hematocrit, hemoglobin and platelets in the blood of hCD34-engrafted MISTRG-6 mice (n=13 (3 donors); n=6 (1 donor)) split by $CD34^+$ cell donors. Data are shown as mean ± SEM. *P* values were calculated using two-tailed, unpaired Mann-Whitney U test (C, D, F) and Kruskal-Wallis with Dunn's correction test (E). * $P < 0.05$, ** $P < 0.01$, *** $P < 0.001$ and **** $P < 0.0001$.

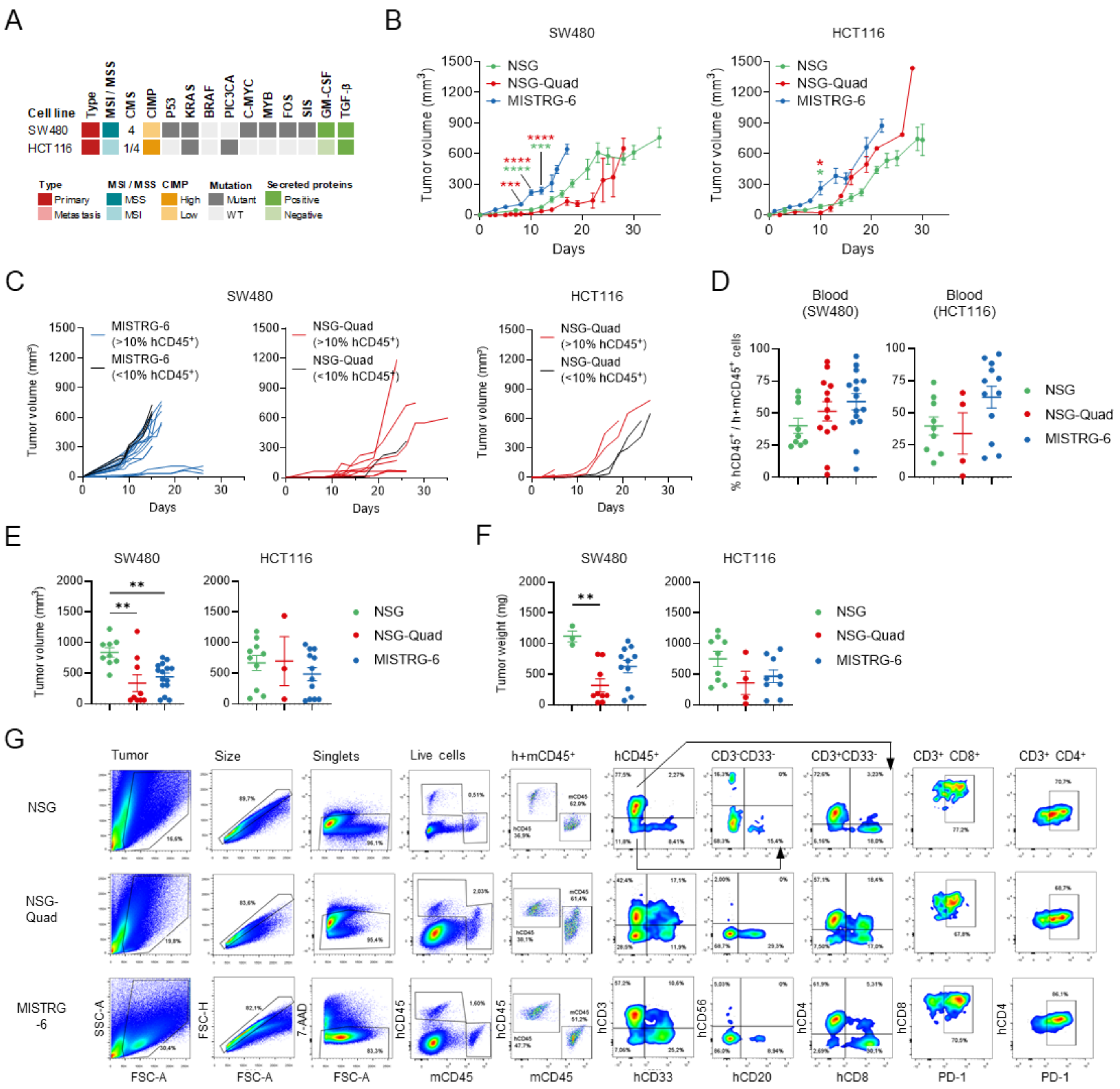

Figure S7: Human immune composition in the blood and tumor of NSG, NSG-Quad and MISTRG-6 humanized mice (related to Figure 5). (**A**) Characteristics of SW480 and HCT116 human CRC cell lines. (**B**) Average tumor growth curves in hCD34-engrafted NSG (n=9), NSG-Quad (n=12) and MISTRG-6 mice (n=15) engrafted with SW480 CRC cells and in NSG (n=10), NSG-Quad (n=4) and MISTRG-6 mice (n=14) engrafted with HCT116 CRC cells. A red asterisk indicates a significant difference between MISTRG-6 and NSG-Quad mice. A green asterisk indicates a significant difference between MISTRG-6 and NSG mice. (**C**) SW480 CRC growth curves in NSG-Quad and MISTRG-6 mice with high (>10%; MISTRG-6: mean=58.97%; NSG-Quad: mean=51.45%) and low (<10%; MISTRG-6: mean=5.89%; NSG-Quad: mean=5.78%) hCD45^{+} cell reconstitution in the blood (left two plots). HCT116 CRC growth curves in NSG-Quad mice with high

(>10%; mean=33.95%) and low (<10%; mean=7.08%) hCD45$^+$ cell reconstitution in the blood (right plot). (**D**) Percentage of hCD45$^+$ cells of total CD45$^+$ cells (mouse and human) in the blood of NSG (n=9 (SW480); n=9 (HCT116)), NSG-Quad (n=13 (SW480); n=4 (HCT116)) and MISTRG-6 humanized mice (n=15 (SW480); n=12 (HCT116)) at 10-15 weeks post engraftment with human cord blood-derived CD34$^+$ cells (end of experiment). (**E**) Tumor volume at the time of analysis (end of experiment) in NSG, NSG-Quad and MISTRG-6 mice. (**F**) Tumor weight at the time of analysis (end of experiment) in NSG, NSG-Quad and MISTRG-6 mice. (**G**) Representative flow cytometry plots showing the gating strategy for identifying human immune cell subsets in the tumor of NSG, NSG-Quad and MISTRG-6 mice. Data are shown as mean ± SEM. *P* values were calculated using one-way ANOVA with Tukey's multiple comparison test. * $P < 0.05$, ** $P < 0.01$, *** $P < 0.001$ and **** $P < 0.0001$. Abbreviations: CIMP, CpG island methylator phenotype; CMS, consensus molecular subtype; MSI, microsatellite instability; MSS, microsatellite stability; WT, wild-type.

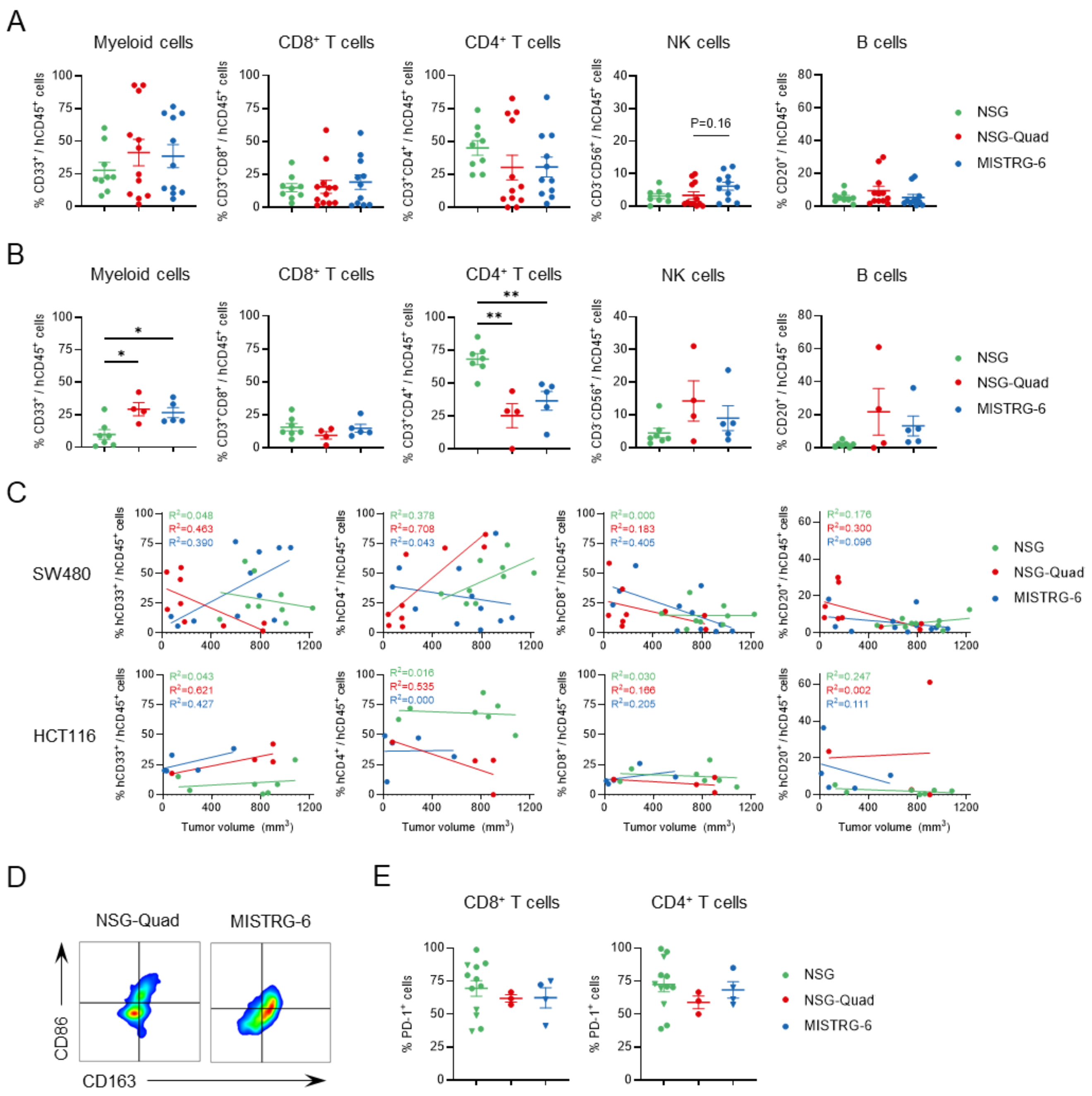

Figure S8: Human immune cell subsets in the tumor of NSG, NSG-Quad and MISTRG-6 humanized mice (related to Figure 5). (**A**) Percentage of human immune cell subsets in the tumor (SW480) of NSG (n=9), NSG-Quad (n=13) and MISTRG-6 humanized mice (n=11). (**B**) Percentage of human immune cell subsets in the tumor (HCT116) of NSG (n=9), NSG-Quad (n=4) and MISTRG-6 humanized mice (n=5). (**C**) Correlation of human immune cell subset frequencies with tumor volume in SW480 and HCT116 CRC xenografts of NSG, NSG-Quad and MISTRG-6 humanized mice. (**D**) Representative flow cytometry plots showing cells expressing CD86 and CD163 in human $CD14^+$ monocytes in SW480 CRC xenografts of NSG-Quad and MISTRG-6 mice. (**E**) Frequency of PD-1-expressing CD8+ T cells and CD4+ T cells in SW480 and HCT116 CRC xenografts of NSG (n=12), NSG-Quad (n=3) and MISTRG-6 humanized mice (n=4). Dots

indicate mice with HCT116 tumors and triangles indicate mice with SW480 tumors. Data are shown as mean ± SEM. *P* values were calculated using one-way ANOVA with Tukey's multiple comparison test. Simple linear regression was performed and R^2 values calculated (C). * $P < 0.05$ and ** $P < 0.01$.

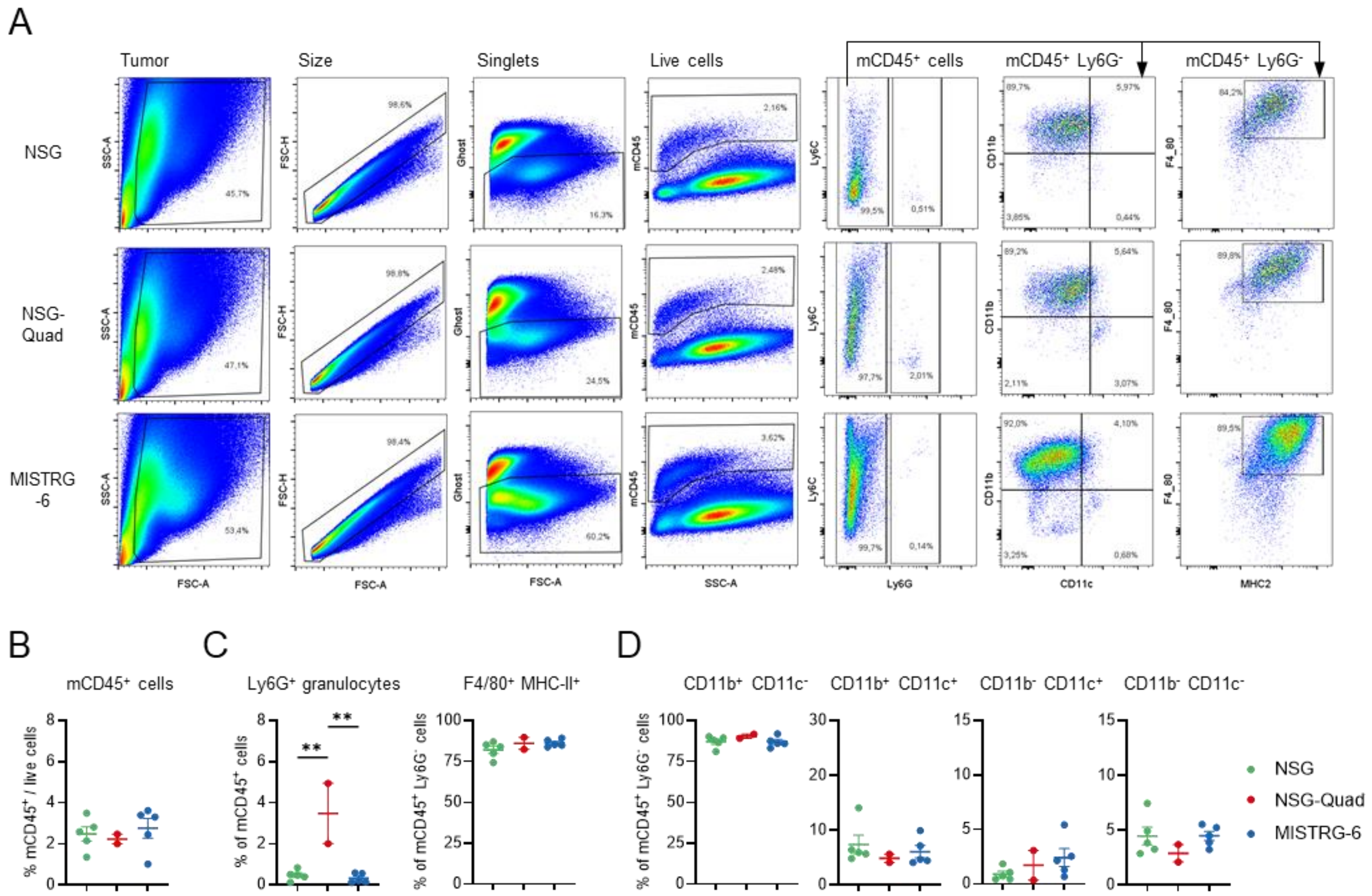

Figure S9: Mouse immune cell subsets in the tumor of NSG, NSG-Quad and MISTRG-6 humanized mice (related to Figure 5). (**A**) Representative flow cytometry plots showing the gating strategy for identifying mouse immune cell subsets in the tumor of NSG, NSG-Quad and MISTRG-6 mice. (**B**) Frequency of mCD45$^+$ cells in tumors of NSG, NSG-Quad and MISTRG-6 humanized mice engrafted with human SW480 CRC cells. (**C**) Frequency of mouse Ly6G$^+$ granulocytes and F4/80$^+$ MHC-II$^+$ macrophages in tumors of NSG, NSG-Quad and MISTRG-6 humanized mice engrafted with human SW480 CRC cells. (**D**) Frequency of four mouse myeloid cell subsets in tumors of NSG, NSG-Quad and MISTRG-6 humanized mice engrafted with human SW480 CRC cells. Data are shown as mean ± SEM. *P* values were calculated using one-way ANOVA with Tukey's multiple comparison test. ** $P < 0.01$.
