# Supplementary material for: Comparison of NSG-Quad and MISTRG-6 humanized mice for modeling circulating and tumor-infiltrating human myeloid cells
Source: Mol Ther Methods Clin Dev. 2025 May 14;33(2):101487. doi: 10.1016/j.omtm.2025.101487 (PMC12152875; doi:10.1016/j.omtm.2025.101487)
Supplement: Document S2. Article plus supplemental information [file mmc2.pdf]

Molecular Therapy
Methods & Clinical Development
Original Article

Comparison of NSG-Quad and MISTRG-6 humanized mice for modeling circulating and tumor-infiltrating human myeloid cells

Anna Chen,[1,4] Viktoria Knöbl,[2,4] Oliver Walzer,[1] Jana Hauser,[1] Ines Neuwirth,[1] Magdalena Frank,[1] Nina Braun,[1] Semina Duvnjak,[1] Johannes Reisecker,[1] Carmen Stecher,[1] Alex Farr,[3] Christine Brostjan,[2] and Dietmar Herndler-Brandstetter[1]

[1]Center for Cancer Research, Medical University of Vienna and Comprehensive Cancer Center, 1090 Vienna, Austria; [2]Department of General Surgery, Division of Vascular Surgery, Medical University of Vienna, 1090 Vienna, Austria; [3]Department of Obstetrics and Gynecology, Division of Obstetrics and Feto-Maternal Medicine, Medical University of Vienna and Comprehensive Center for Pediatrics (CCP), Medical University of Vienna, 1090 Vienna, Austria

Humanized mice are valuable preclinical models for immuno-oncology research because they allow modeling of human immune cells and human tumors *in vivo*. Myeloid cells are highly abundant in many tumors and have been associated with tumor progression, metastasis, and therapy resistance. Next-generation humanized mice have been generated to improve the development, diversity, and function of human myeloid cells. In this study, we analyzed human immune cell development and myeloid cell composition in NSG-Quad and MISTRG-6 mice. NSG-Quad mice supported the development of tissue-resident and tumor-infiltrating human macrophages at levels almost comparable to those of MISTRG-6 mice. However, the development of human CD4$^+$ and CD8$^+$ T cells was impaired in the blood and spleen but not in the tumor of NSG-Quad mice. In a subset of NSG-Quad mice, human monocytes exhibited increased cellular granularity and elevated expression of activation and checkpoint molecules, consistent with a monocyte hyperactivation syndrome. Our study provides a comprehensive comparative analysis of the frequency and characteristics of circulating, tissue-resident, and tumor-infiltrating myeloid cell populations in NSG-Quad and MISTRG-6 mice, which is key to accurately design and interpret human tumor xenograft studies, particularly with regard to faithful reconstruction of the human tumor-immune microenvironment and preclinical testing.

INTRODUCTION

The field of onco-immunology is rapidly evolving and thereby transforming the drug development and treatment landscape. However, only a small fraction of patients benefits from cancer immunotherapy, and the factors underlying successful immunotherapy are still not fully understood. Thus, there is a need to develop preclinical models that recapitulate the human tumor-immune microenvironment and allow meaningful testing of novel immunotherapies or rational combinatorial strategies, including myeloid cell-targeted therapies. Myeloid cells have been shown to promote tumor growth by stimulating angiogenesis, enhancing tumor cell migration and invasion, suppressing antitumor immune responses, and promoting resistance to immunotherapies.[1–3] In colorectal cancer (CRC), tumor-infiltrating myeloid cells, in particular M2 macrophages, have been associated with poor prognosis,[4] and only patients who have a mismatch repair-deficient (MMRd) microsatellite instability (MSI) phenotype show partial or complete responses to anti-PD-1 immune checkpoint inhibition.[5,6] In CRC patients with a mismatch repair-proficient (MMRp) microsatellite stable (MSS) phenotype, myeloid cells are the most abundant immune cells in the tumor microenvironment and therefore represent an attractive therapeutic target.

Humanized mice, defined as immunodeficient mice co-engrafted with a human tumor and a human immune system, have provided valuable information for human-specific drug testing, including anti-PD-1 antibody and chimeric antigen receptor (CAR) T cell therapy.[7–9] More than 50 humanized mouse models have been developed, including the commonly used non-obese diabetic (NOD) *Prkdc*^{scid} *Il2rg*$^{-/-}$ (NSG) mice and Balb/c x 129 *Rag2*$^{-/-}$ *Il2rg*$^{-/-}$ (BRG) mice.[8] Reconstitution of a human immune system is achieved by transplantation of human CD34$^+$ hematopoietic stem and progenitor cells (HSPCs) into immunodeficient mice. However, to achieve faithful reconstitution of a human immune system, next-generation humanized mice have been developed, which express human cytokines that support the development of a diverse and functional human immune system.[10] Among others, interleukin-3 (IL-3), IL-6, granulocyte-macrophage colony-stimulating factor (GM-CSF) and macrophage colony-stimulating factor

Received 7 October 2024; accepted 12 May 2025;
https://doi.org/10.1016/j.omtm.2025.101487.

[4]These authors contributed equally

Correspondence: Dietmar Herndler-Brandstetter, Center for Cancer Research, Medical University of Vienna and Comprehensive Cancer Center, 1090 Vienna, Austria.
E-mail: dietmar.herndler-brandstetter@meduniwien.ac.at

© 2025 The Author(s).
Published by Elsevier Inc. on behalf of The American Society of Gene and Cell Therapy.
This is an open access article under the CC BY license (http://creativecommons.org/licenses/by/4.0/).

(M-CSF) have been shown to be important for functional multilineage development of the myeloid cell compartment, especially monocyte subsets and tissue-resident macrophages.[11] In particular, M-CSF has been shown to promote monocyte to macrophage differentiation, macrophage survival, and proliferation as well as to support the development of tissue-resident and tumor-associated macrophages. In addition, M-CSF primes macrophages for a variety of immune functions, including phagocytosis, secretion of cytokines, and angiogenesis via production of vascular endothelial growth factor (VEGF).[12]

In order to improve human myeloid lineage development and function, next-generation humanized mouse models that express human M-CSF have been developed. MISTRG-6 mice are BRG mice that express human M-CSF, IL-3/GM-CSF, signal regulatory protein alpha (SIRPα), thrombopoietin (THPO), and IL-6 in a human gene knock-in and mouse gene knock-out manner (Figure S1A).[11,13] MISTRG-6 mice support the development and function of human monocytes, macrophages, and natural killer (NK) cells, including infiltration of human melanoma xenografts by human macrophages.[11,14,15] Quadruple transgenic NSG-Quad mice are NSG mice that express human IL-3, GM-CSF, stem cell factor (SCF), and M-CSF (Figure S1A). NSG-Quad mice support human $CD33^+$ and $CD14^+$ cell development and can be engrafted with human-induced pluripotent stem cells.[16,17]

However, it has not been investigated whether humanized NSG-Quad mice support the development of different human myeloid cell lineages, including monocyte subtypes as well as tissue-resident and tumor-infiltrating macrophages. Understanding mouse strain-specific differences, particularly with respect to the human myeloid cell compartment, is key for faithful modeling of the human tumor-immune microenvironment and for preclinical testing of cancer immunotherapies. We therefore performed an in-depth analysis of NSG-Quad mice, compared them to other humanized mouse strains, and investigated the ability of NSG-Quad mice to model circulating, tissue-resident and tumor-infiltrating human myeloid cell populations.

RESULTS

Characterization of NSG-Quad and MISTRG-6 mice

Whereas NSG-Quad mice express human IL-3, GM-CSF, SCF as homozygous transgenes and human M-CSF as heterozygous or homozygous transgene, MISTRG-6 mice express human IL-3, GM-CSF, THPO, and M-CSF as homozygous knock-in genes and human SIRPA and IL-6 as heterozygous knock-in genes. To assess human cytokine production in these mouse strains, we measured human M-CSF, GM-CSF, and IL-6 in the plasma of untreated NSG, NSG-Quad, and MISTRG-6 mice using ELISA. Human M-CSF protein levels in the plasma were higher in MISTRG-6 compared to NSG-Quad mice, whereas human M-CSF was not detected in NSG mice (Figure S1B). Because the bone marrow is the major site of myeloid cell development, we quantified human M-CSF, GM-CSF, and IL-6 in the supernatant of untreated and lipopolysaccharide (LPS)-treated bone marrow cells. Our results demonstrate that human M-CSF secretion by bone marrow cells was comparable between NSG-Quad and MISTRG-6 mice (Figure S1C).

Next, we used a Vet abc hematology analyzer to assess blood cell parameters and quantify mouse white blood cells. Mouse white blood cells, in particular monocytes and granulocytes, were higher in MISTRG-6 mice compared to NSG and NSG-Quad mice, whereas platelets were lower in MISTRG-6 mice compared to NSG and NSG-Quad mice (Figures S1D–S1F). In addition, mouse $CD11b^+CD11c^+$ dendritic cells were reduced, whereas $CD11b^-CD11c^-$ cells were slightly increased in MISTRG-6 compared to NSG-Quad mice (Figure S1G).

Human immune cell development in NSG-Quad and MISTRG-6 humanized mice

To evaluate the efficacy of human immune cell reconstitution, newborn NSG, NSG-Quad, and MISTRG-6 mice were transplanted intrahepatically with cord-blood-derived human $CD34^+$ HSPCs (Figure S2A). At week 8 post-engraftment, the frequency of human $CD45^+$ ($hCD45^+$) cells in the blood was comparable between the three humanized mouse models (Figures 1A and S2B), indicating suitability of the used engraftment protocols. At week 10–15 post-engraftment, $hCD45^+$ cell reconstitution increased in all mouse models, with a significantly higher frequency of $hCD45^+$ cells in MISTRG-6 compared to NSG mice (Figure 1A). Engraftment levels in the bone marrow were comparable between the three humanized mouse strains (Figure S3A). At week 8 post-engraftment, all three mouse models showed development of human immune cells in the peripheral blood, including human $CD4^+$ T cells, $CD8^+$ T cells, $CD20^+$ B cells, $CD33^+$ myeloid cells, and $CD3^-CD56^+$ NK cells (Figures 1B, 1C, and S2B). Human CD33 was used to detect myeloid progenitors, monocytes, granulocytes, dendritic cells, and mast cells. In contrast to CD11b, CD33 is more exclusively expressed on myeloid cells. NSG-Quad and MISTRG-6 mice, which express human M-CSF, showed a significantly improved development of $hCD33^+$ myeloid cells compared to NSG mice (Figure 1C). At week 10–15 post-engraftment, $hCD33^+$ myeloid cells dominated the human immune cell pool in the blood of NSG-Quad mice (Figure 1D). In contrast to MISTRG-6 mice,[11,15] the high frequency of human myeloid cells in NSG-Quad was not able to support human NK cells in the circulation at week 8 and week 10–15 post-engraftment (Figures 1C and 1D). In the spleen, NSG-Quad mice had a decreased $hCD45^+$ cell engraftment (mean: 65% of $h+mCD45^+$ cells) but a very high frequency of $hCD33^+$ myeloid cells (mean: 33% of $hCD45^+$ cells) compared to NSG and MISTRG-6 mice (Figures S3B–S3D). In contrast to the blood, the frequency of NK cells in the spleen of NSG-Quad mice was significantly increased compared to NSG mice but still 2-fold lower than in MISTRG-6 mice (Figure S3D). NSG-Quad displayed a severe defect in the development of human $CD4^+$ and $CD8^+$ T cells in the blood and spleen compared to NSG and MISTRG-6 mice (Figures 1C, 1D, and S3D). Human $CD45^+$ cell engraftment was comparable when using either one or three donors for human $CD34^+$ HSPCs (Figure S3E).

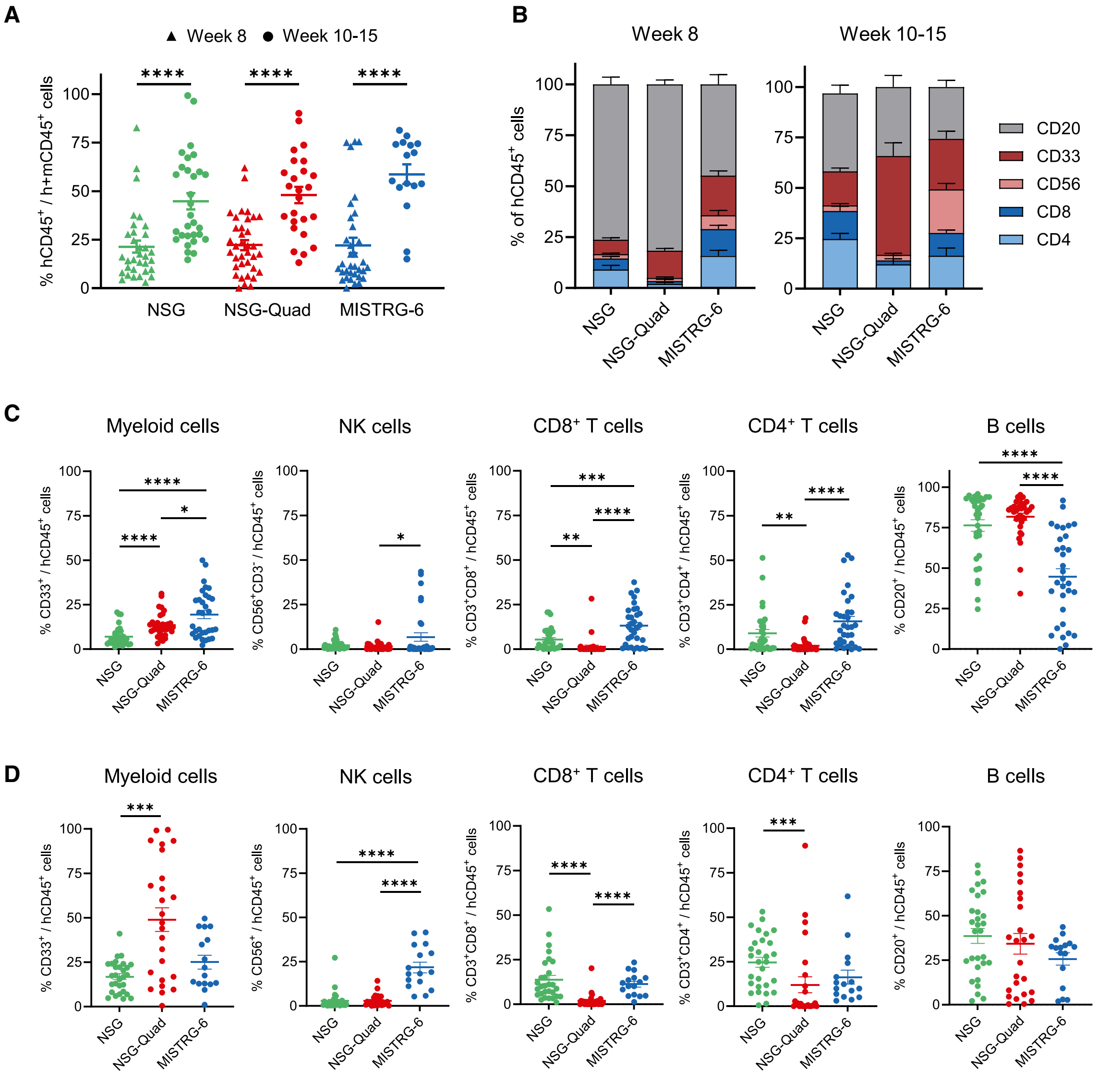

Figure 1. NSG-Quad mice support human hematopoietic cell engraftment and multilineage immune cell development

(A) Percentage of human CD45$^+$ (hCD45$^+$) cells of total CD45$^+$ cells (mouse and human) in the blood at 8 weeks and 10–15 weeks post-engraftment with human-cord-blood-derived CD34$^+$ cells. (B) Human immune cell composition in the blood of NSG (n = 33), NSG-Quad (n = 34), and MISTRG-6 mice (n = 32) 8 weeks post-engraftment and in the blood of NSG (n = 29), NSG-Quad (n = 25), and MISTRG-6 mice (n = 16) 10–15 weeks post-engraftment. B cells (CD3$^-$CD56$^-$CD33$^-$CD20$^+$), myeloid cells (CD3$^-$CD56$^-$CD20$^-$CD33$^+$), NK cells (CD3$^-$CD56$^+$), CD8$^+$ T cells (CD3$^+$CD4$^-$CD8$^+$), and CD4$^+$ T cells (CD3$^+$CD8$^-$CD4$^+$). (C) Percentage of human immune cell subsets in the blood 8 weeks post-engraftment (data from B). (D) Percentage of human immune cell subsets in the blood 10–15 weeks post-engraftment (data from B). Data are shown as mean ± SEM. p values were calculated using one-way or two-way ANOVA (A) with Tukey's multiple comparison test. *p < 0.05, **p < 0.01, ***p < 0.001, and ****p < 0.0001.

The use of three donors increased CD4$^+$ T cell frequency in NSG-Quad and MISTRG-6 mice (Figure S3F).

Next, we compared the impact of heterozygous and homozygous expression of the human M-CSF transgene on human immune cell composition in NSG-Quad mice. NSG-Quad$^{+/+}$ (homozygous for human M-CSF) and NSG-Quad$^{+/-}$ (heterozygous for human M-CSF) mice had comparable hCD45$^+$ cell engraftment in the blood. NSG-Quad$^{+/+}$ mice had an increased frequency of circulating human NK cell in the blood but lacked circulating CD4$^+$ and CD8$^+$ T cells (Figures 2A–2C). We also investigated the direct impact of human M-CSF on human immune cell composition by comparing NSG-Quad and NSGS mice, which differ only in their expression of the human M-CSF transgene (Figure S1A). Human CD45$^+$ cell engraftment in the blood was comparable between NSG-Quad and NSGS mice at week 8 and 10–15

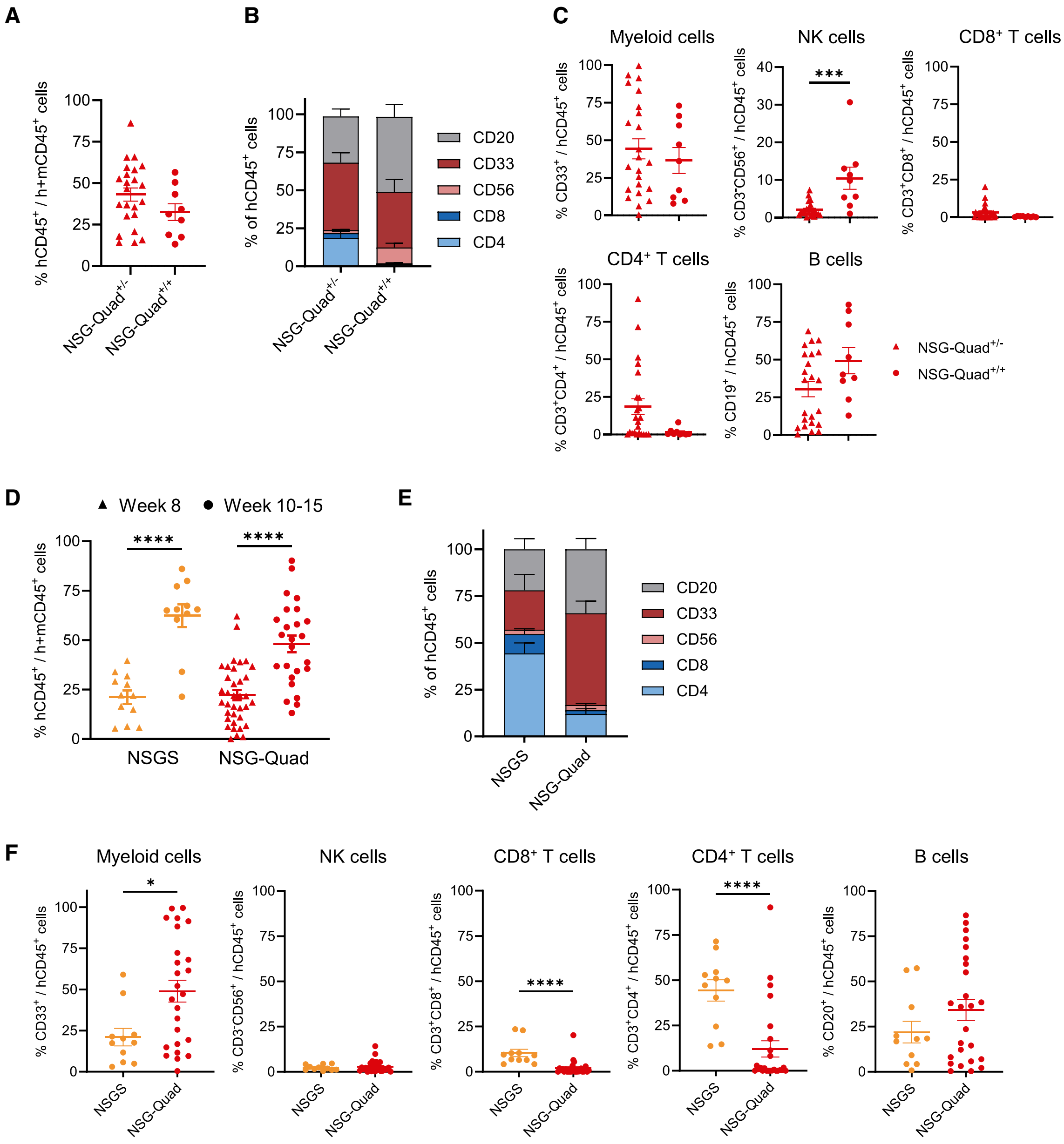

Figure 2. NSG-Quad mice support the development of human myeloid cells

(A) Percentage of human CD45$^+$ (hCD45$^+$) cells of total CD45$^+$ cells (mouse and human) in the blood of NSG-Quad$^{+/-}$ mice (heterozygous M-CSF, $n = 24$) and NSG-Quad$^{+/+}$ mice (homozygous M-CSF, $n = 9$) at 10–15 weeks post-engraftment with human-cord-blood-derived CD34$^+$ cells. (B) Human immune cell composition in the blood of NSG-Quad$^{+/-}$ mice ($n = 24$) and NSG-Quad$^{+/+}$ mice ($n = 9$) 10–15 weeks post-engraftment. (C) Percentage of human immune cell subsets in the blood of NSG-Quad$^{+/-}$ and NSG-Quad$^{+/+}$ mice 10–15 weeks post-engraftment (data from B). (D) Percentage of human CD45$^+$ (hCD45$^+$) cells of total CD45$^+$ cells (mouse and human) in the blood at 8 weeks and 10–15 weeks post-engraftment with human-cord-blood-derived CD34$^+$ cells. (E) Human immune cell composition in the blood of NSGS ($n = 11$) and NSG-Quad mice ($n = 25$) 10–15 weeks post-engraftment. (F) Percentage of human immune cell subsets in the blood of NSGS and NSG-Quad mice 10–15 weeks post-engraftment (data from E). Data are shown as mean ± SEM. p values were calculated using two-tailed, unpaired Student's t test (A), two-way ANOVA with Tukey's multiple comparison test (D) and two-tailed, unpaired Mann-Whitney U test (C, F). *$p < 0.05$, ***$p < 0.001$, and ****$p < 0.0001$.

post-engraftment (Figure 2D). However, NSG-Quad mice displayed an increased frequency of CD33$^+$ myeloid cells and a reduced frequency of CD4$^+$ and CD8$^+$ T cells in the blood and spleen (Figures 2E, 2F, and S4A–S4D). The frequency of human NK cells was increased in the spleen but not in the blood of NSG-Quad mice compared to NSGS mice (Figure S4D).

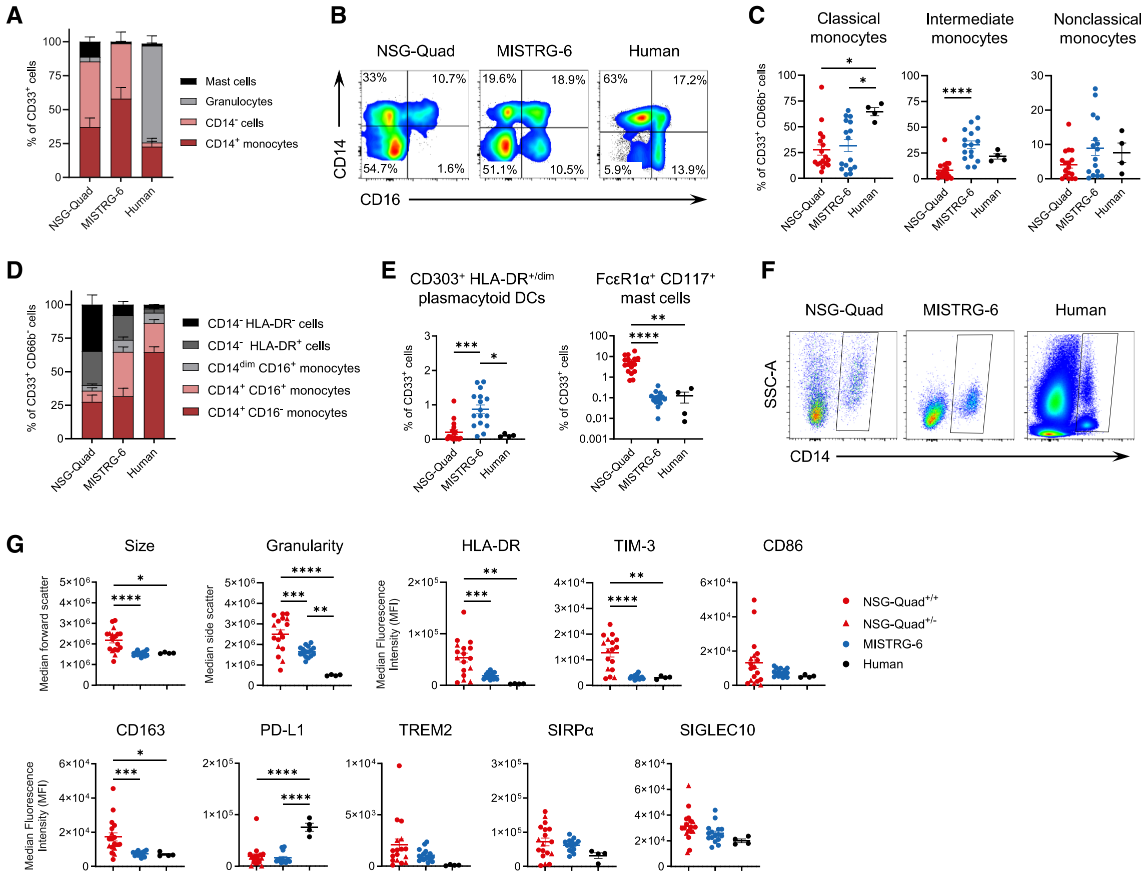

Figure 3. NSG-Quad mice support the development of diverse human myeloid cell populations, but monocytes exhibit a hyperactivated phenotype

(A) Frequency of CD14$^+$ monocytes, CD66b$^+$ granulocytes, FcεR1α$^+$CD117$^+$ mast cells, and CD33$^+$CD14$^-$ myeloid cells in the blood of NSG-Quad (n = 19), MISTRG-6 (n = 11), and healthy human adults (n = 4). (B) Representative flow cytometry plots showing cells expressing CD14 and CD16 within human CD33$^+$CD66b$^-$ myeloid cells in the blood of NSG-Quad, MISTRG-6, and human adults. (C) Frequency of CD14$^+$CD16$^-$ classical, CD14$^+$CD16$^+$ intermediate, and CD14^{dim}CD16$^+$ non-classical monocytes in the blood of NSG-Quad (n = 17), MISTRG-6 (n = 16), and human adults (n = 4). (D) Composition of the human CD33$^+$CD66b$^-$ myeloid cell population based on the expression of CD14, CD16, and HLA-DR in NSG-Quad (n = 17), MISTRG-6 (n = 16), and human adults (n = 4). (E) Frequency of CD303$^+$HLA-DR$^{+/dim}$ plasmacytoid dendritic cells (pDCs) and FcεR1α$^+$CD117$^+$HLA-DR$^-$SIRPA$^-$ mast cells in the blood of NSG-Quad, MISTRG-6, and human adults. (F) Representative flow cytometry plots showing hCD45$^+$ live cells in the blood of NSG-Quad, MISTRG-6, and human adults. (G) Median forward scatter (cell size), median side scatter (granularity), and median fluorescence intensity (MFI) of eight key myeloid molecules on human CD14$^+$ monocytes in the blood of NSG-Quad (n = 18), MISTRG-6 (n = 16), and human adults (n = 4). Data are shown as mean ± SEM. p values were calculated using Kruskal-Wallis with Dunn's correction test (C, E) and one-way ANOVA with Tukey's multiple comparison test (G). *$p < 0.05$, **$p < 0.01$, ***$p < 0.001$, and ****$p < 0.0001$.

Development of human myeloid cell subsets in NSG-Quad and MISTRG-6 humanized mice

Next, we analyzed the human myeloid cell compartment in the blood of NSG-Quad humanized mice using an 18-color flow cytometry panel and compared it to MISTRG-6 and human peripheral blood. The gating strategy for the different human myeloid cell subsets is shown in Figures S5A–S5C. Our results demonstrate that a variety of human myeloid cell subpopulations developed in NSG-Quad mice (Figures 3A and S6A). All three subsets of human monocytes could be detected in the blood of NSG-Quad mice. However, NSG-Quad mice had less CD14$^+$CD16$^+$ intermediate monocytes and slightly lower CD14^{dim}CD16$^+$ nonclassical monocytes (p = 0.08) compared to MISTRG-6 mice (Figures 3B and 3C).

The CD33$^+$ myeloid compartment in the blood of NSG-Quad mice was dominated by CD14$^-$ cells including CD14$^-$HLA-DR$^+$ dendritic cells (Figure 3D). Interestingly, CD303$^+$HLA-DR$^{+/dim}$ plasmacytoid dendritic cells (pDCs) were less abundant in the blood of NSG-Quad compared to MISTRG-6 mice, whereas the frequency of circulating FcεR1α$^+$CD117$^+$ mast cells, which lacked expression of HLA-DR and SIRPα, was 55-fold higher in NSG-Quad compared to MISTRG-6 mice and humans (Figures 3E and S6B). To better characterize the human myeloid cell subpopulations in NSG-Quad and MISTRG-6 humanized mice, we analyzed cell size, granularity, and eight key myeloid molecules (HLA-DR, CD86, CD163, PD-L1, TREM2, SIRPA, TIM-3, and SIGLEC10). CD14$^+$ monocytes in NSG-Quad mice showed increased cell size and granularity as well as increased expression of

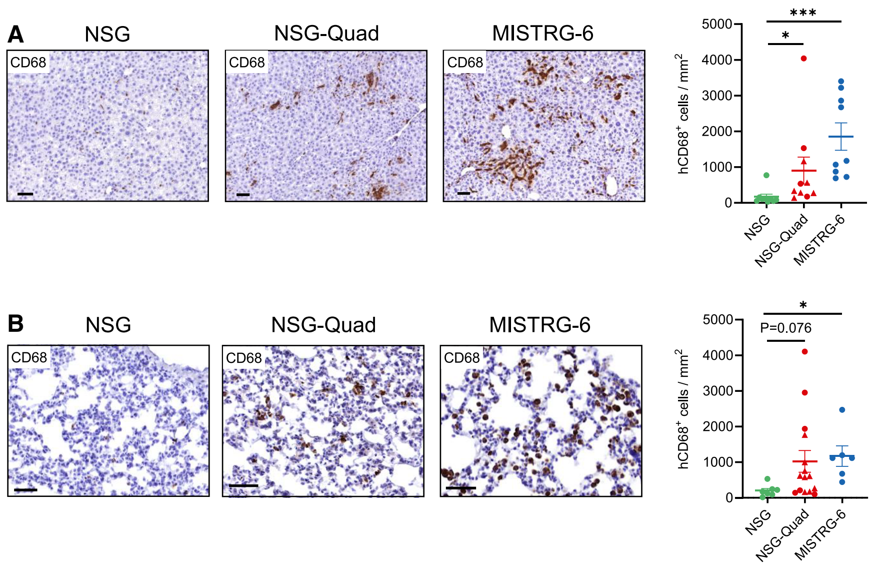

Figure 4. NSG-Quad mice support the development of tissue-resident human macrophages

(A) IHC pictures and dot plot graph show human $CD68^+$ macrophages in the liver of NSG (n = 9), NSG-Quad (n = 10), and MISTRG-6 mice (n = 9). Bar scale: 50 μm. Triangle symbols represent NSG-$Quad^{+/-}$ mice. (B) IHC pictures and dot plot graph show human $CD68^+$ macrophages in the lung of NSG (n = 7), NSG-Quad (n = 15), and MISTRG-6 mice (n = 6). Bar scale: 50 μm. Triangle symbols represent NSG-$Quad^{+/-}$ mice. Data are shown as mean ± SEM. p values were calculated using Kruskal-Wallis with Dunn's correction test. *$p < 0.05$ and ***$p < 0.001$.

HLA-DR, TIM-3, and CD163 compared to MISTRG-6 mice (Figures 3F and 3G). This is consistent with a monocyte hyperactivation syndrome that has also been reported to occur in a subgroup of NSGS mice.[18,19] To identify which myeloid subpopulations are impacted by hyperactivation in NSG-Quad mice, we analyzed cellular characteristics and the expression of eight key myeloid markers across six myeloid cell types in the circulation. While granulocytes and $CD33^+CD14^-CD66b^-$ myeloid cells were comparable between NSG-Quad and MISTRG-6 mice, classical monocytes, intermediate monocytes, pDCs, and mast cells showed an increase in cell size, granularity, and an elevated expression of multiple activation and checkpoint molecules in NSG-Quad mice (Figures S6C and S6D). In particular, SIGLEC10, TIM-3, and CD163 were increased in pDCs and mast cells of NSG-Quad mice, whereas the expression of PD-L1 and TREM2 on myeloid cell subsets was comparable between NSG-Quad and MISTRG-6 mice.

Since human M-CSF also enhances the phagocytic capacity of mature human macrophages, these human $SIRP\alpha^+$ macrophages can phagocytose mouse erythrocytes, which do not express human CD47, the inhibitory ligand of SIRPα.[11,20,21] We therefore analyzed red blood cell (RBC) counts as well as hematocrit and hemoglobin levels using EDTA-coated tubes and the Vet abc hematology system. Our results demonstrate that both next-generation humanized mice that support human myeloid cell development have a lower RBC count, a decreased hematocrit, and lower hemoglobin and platelet levels compared to NSG mice at 10–15 weeks post-engraftment (Figure S6E). Whether M-CSF was expressed heterozygous or homozygous in NSG-Quad mice did not make a difference (Figure S6E). Similarly, whether one $CD34^+$ donor or three $CD34^+$ donors were used did not significantly affect hemoglobin levels in MISTRG-6 mice (Figure S6F).

Development of tissue-resident human macrophages in NSG-Quad and MISTRG-6 humanized mice

To assess whether tissue-resident macrophages develop in NSG-Quad mice, we performed immunohistochemistry on formalin-fixed, paraffin-embedded (FFPE) liver and lung samples. Whereas tissue-resident human $CD68^+$ macrophages were virtually absent in the liver and lung of NSG mice, $CD68^+$ cells could be detected in NSG-Quad and MISTRG-6 mice (Figures 4A and 4B). This indicates that transgenic expression of human M-CSF in NSG-Quad mice supports the development of tissue-resident human macrophages at levels almost comparable to MISTRG-6 mice.

Tumor-infiltrating human myeloid cell subsets in NSG-Quad and MISTRG-6 humanized mice

Humanized mouse models have become valuable preclinical tools for immuno-oncology research. Because the tumor-immune microenvironment plays an important role, we analyzed tumor growth and infiltration of human immune cell subsets into human tumor xenografts in NSG-Quad and MISTRG-6 mice. We used two well-established CRC xenograft models, SW480 and HCT116, which have a distinct MSS/MSI and mutation profile (Figure S7A). Human CRC cells were injected subcutaneously, and caliper measurement was performed to calculate the tumor volume.

Our results demonstrate that CRC growth was accelerated during the early phase in MISTRG-6 compared to NSG-Quad and NSG humanized mice and was more pronounced in SW480 than HCT116 tumors (Figures 5A and S7B). Notably, MISTRG-6 mice with a very low $hCD45^+$ cell engraftment displayed a similar fast SW480 growth rate compared to MISTRG-6 mice with a high $hCD45^+$ engraftment level (Figure S7C). Overall, the NSG, NSG-Quad, and MISTRG-6 mice used for tumor xenograft experiments had comparable $hCD45^+$ cell engraftment in the blood (Figure S7D). Yet, more $hCD45^+$ cells infiltrated SW480 tumors in MISTRG-6 mice compared to NSG and NSG-Quad mice (Figure 5B), although tumor volume and tumor weight at the time of analysis (endpoint) did not significantly differ between NSG-Quad and MISTRG-6 mice (Figures S7E and S7F). The peripheral blood immune cell composition differed significantly from the immune cell composition in the tumor, with more $CD4^+$ T cells and less $CD20^+$ B cells infiltrating SW480 and HCT116 tumors (Figures 5C, 5D, and S7G). NSG-Quad and MISTRG-6 mice displayed a higher frequency of

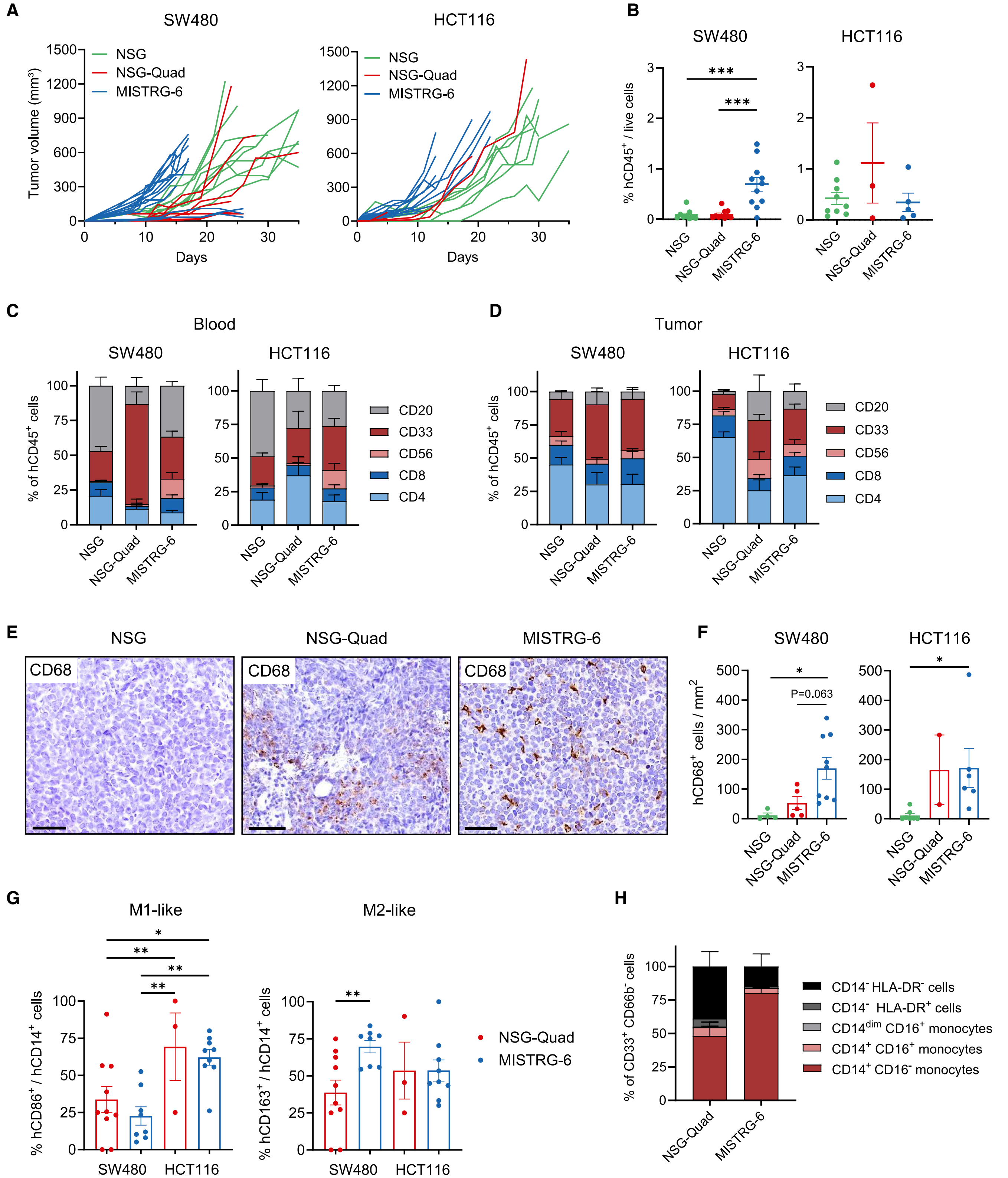

(legend on next page)

tumor-infiltrating myeloid cells compared to NSG mice, whereas NSG-Quad and MISTRG-6 mice had less tumor-infiltrating $CD4^+$ T cells compared to NSG mice (Figures S8A and S8B). Because the tumor volume varied between individual mice, we also analyzed the frequency of immune cells in correlation with the tumor volume. The frequency of human myeloid cells, T cells, and B cells did not differ between small (<300 mm^3) and large (>600 mm^3) HCT116 tumors (Figure S8C). However, small SW480 tumors were infiltrated with a higher frequency of $CD33^+$ myeloid cells and $CD20^+$ B cells and a lower frequency of $CD4^+$ T cells compared to large SW480 tumors in NSG-Quad mice (Figure S8C).

Immunohistochemical analysis revealed a high number of human $CD68^+$ macrophages infiltrating SW480 and HCT116 tumors of NSG-Quad and MISTRG-6 mice, whereas human tumor-associated macrophages (TAMs) were virtually absent in NSG mice (Figures 5E and 5F). Next, we analyzed TAM subset distribution using CD86 as a marker for M1-like TAMs and CD163 for pro-tumorigenic M2-like TAMs. The frequency of $CD86^+$ M1-like TAMs was higher in HCT116 tumors of both NSG-Quad and MISTRG-6 mice, which may be due to the MSI status of HCT116 tumors (Figure 5G). In contrast, the frequency of CD163-expressing M2-like TAMs was highest in SW480 tumors in MISTRG-6 mice (Figures 5G and S8D), which may be due to the MSS status and high secretion of GM-CSF by SW480 tumors (Figure S7A). Next, we characterized the myeloid cell compartment in the tumor xenografts in more detail. Similar to the blood, SW480 CRC xenografts in NSG-Quad mice exhibited a higher frequency of human $CD33^+CD66b^-CD14^-HLA\text{-}DR^-$ cells compared to MISTRG-6 mice (Figure 5H). In contrast, the myeloid compartment in SW480 CRC xenografts of MISTRG-6 mice was dominated by $CD14^+CD16^-$ classical monocytes (Figure 5H). Flow cytometric assessment of the T cell activation marker programmed cell death protein 1 (PD-1), an important target of immune checkpoint inhibition, revealed that PD-1 expression in tumor-infiltrating $CD8^+$ and $CD4^+$ T cells was comparable between NSG, NSG-Quad, and MISTRG-6 mice (Figure S8E).

Because the frequency of circulating mouse myeloid cell subsets was different between NSG-Quad and MISTRG-6 mice and may affect tumor growth and the tumor microenvironment (Figures S1D–S1G), we assessed the frequency of mouse $CD45^+$ cells and myeloid cell subsets infiltrating the human CRC tumor xenografts. About 2%–4% of all live cells in the tumor were mouse $CD45^+$ cells (Figures S9A and S9B). The vast majority of $mCD45^+$ cells (>80%) displayed an $F4/80^+$ MHC II^+ TAM phenotype. Tumors in NSG-Quad mice exhibited greater infiltration of mouse $Ly6G^+$ granulocytes compared to NSG and MISTRG-6 mice, whereas the abundance of TAMs and four myeloid subsets, defined by differential expression of mouse CD11b and CD11c, did not differ between the three mouse strains (Figure S9C).

In summary, NSG-Quad mice supported the development of different human myeloid cell lineages and promoted the development of tissue-resident as well as tumor-infiltrating human $CD68^+$ macrophages at a level almost comparable to MISTRG-6 mice. However, the development of NK cells, $CD4^+$ T cells, and $CD8^+$ T cells in NSG-Quad mice was reduced compared to MISTRG-6 mice.

DISCUSSION

In this study, we analyzed human immune cell development in next-generation humanized NSG-Quad mice, in particular with respect to modeling circulating and CRC-infiltrating human myeloid cell populations. By using 10- and 18-color flow cytometry panels, we showed that expression of human M-CSF in both NSG-Quad and MISTRG-6 humanized mice improved $CD33^+$ myeloid cell development in the blood compared to NSG and NSGS mice. However, the development of intermediate and nonclassical monocytes, which are important for antigen presentation and complement-/FcR-mediated phagocytosis, respectively, was more efficient in MISTRG-6 compared to NSG-Quad mice. Furthermore, the human $CD14^+$ monocytes that developed in NSG-Quad mice displayed unusually high cellular granularity and expression of activation (HLA-DR) and checkpoint molecules (TIM-3). In the spleen, NSG-Quad mice had a 4.6-fold expansion of $CD33^+$ myeloid cells compared to NSGS mice, whereas the frequency of human $CD4^+$ and $CD8^+$ T cells was severely impaired in NSG-Quad compared to NSGS mice.

The differences observed between NSG-Quad and MISTRG-6 mice may, in part, be related to transgenic (over)expression versus physiological knock-in expression of human GM-CSF and M-CSF. The increased cell size and granularity, along with elevated expression of activation and checkpoint molecules HLA-DR, TIM-3, and

Figure 5. NSG-Quad mice promote the development of tumor-infiltrating human macrophages

(A) Tumor growth curves in NSG (n = 9), NSG-Quad (n = 12), and MISTRG-6 mice (n = 15) engrafted with SW480 CRC cells and in NSG (n = 10), NSG-Quad (n = 4), and MISTRG-6 mice (n = 14) engrafted with HCT116 CRC cells. (B) Frequency of $hCD45^+$ cells in the tumor of NSG, NSG-Quad, and MISTRG-6 humanized mice engrafted with SW480 or HCT116 CRC cells (10–15 weeks post-engraftment with human-cord-blood-derived $CD34^+$ cells; end of experiment). (C) Human immune cell composition in the blood of NSG, NSG-Quad, and MISTRG-6 humanized mice engrafted with SW480 or HCT116 CRC cells (10–15 weeks post-engraftment with human-cord-blood-derived $CD34^+$ cells; end of experiment; data from Figure S7G). (D) Human immune cell composition in the tumor xenografts of NSG (n = 9, SW480; n = 10, HCT116), NSG-Quad (n = 10, SW480; n = 3, HCT116), and MISTRG-6 mice (n = 15, SW480; n = 12, HCT116). (E) Representative IHC pictures show SW480 CRC-infiltrating human $CD68^+$ macrophages in NSG, NSG-Quad, and MISTRG-6 humanized mice. Scale bar: 50 μm. (F) Frequency of human $CD68^+$ macrophages in CRC xenografts of NSG (n = 11), NSG-Quad (n = 7), and MISTRG-6 mice (n = 15) analyzed by IHC. (G) Frequency of $CD86^+$ (M1-like) and $CD163^+$ (M2-like) human $CD14^+$ monocytes in SW480 and HCT116 CRC xenografts of NSG-Quad (n = 13) and MISTRG-6 mice (n = 17). (H) Composition of the human $CD33^+CD66b^-$ myeloid cell population based on the expression of CD14, CD16, and HLA-DR in SW480 CRC xenografts of NSG-Quad (n = 8) and MISTRG-6 (n = 3) mice. Data are shown as mean ± SEM. p values were calculated using one-way or two-way ANOVA (G) with Tukey's multiple comparison test. $^*p < 0.05$, $^{**}p < 0.01$, and $^{***}p < 0.001$.

CD163 in human $CD14^+$ monocytes, indicate a monocyte hyperactivation syndrome in a subset of $CD34^+$-cell-engrafted NSG-Quad mice. A monocyte hyperactivation syndrome has also been observed in a subset of NSGS mice.[18,19] Interestingly, pDCs and mast cells also exhibited increased cell size and granularity, indicative of increased cytoplasmic protein content and an activated cell state. This was accompanied by increased expression of immunomodulatory molecules (TIM-3, SIGLEC10, and TREM2), phagocytosis-associated markers (SIRPA and TREM2), and the M2-type marker CD163. It will therefore be important to identify and exclude those NSG-Quad mice that display features of a monocyte hyperactivation syndrome, in order to not confound accurate interpretation of human tumor xenograft and human tumor-immune microenvironment studies. Human M-CSF promotes the development and maturation of human phagocytic monocytes and macrophages, which appears to drive the low RBC count observed in $CD34^+$-cell-engrafted NSG-Quad and MISTRG-6 mice, and restricts the time window for tumor xenograft studies. Notably, 67% of NSG-Quad and 20% of MISTRG-6 mice displayed slow SW480 tumor growth. All of these mice showed >10% $hCD45^+$ engraftment in the blood, but slow-growing SW480 tumors in NSG-Quad mice showed a particularly high infiltration with $CD33^+$ myeloid cells. A systemic monocyte activation syndrome in NSG-Quad mice may have contributed to the slow tumor growth, and the occurrence of severe anemia in the three NSG-Quad and three MISTRG-6 mice with slow tumor growth prevented us to continue the experiments beyond the 26 days post-tumor challenge. The increased tumor growth in MISTRG-6 mice may be due to the high number of tumor-infiltrating $CD163^+$ human macrophages. These macrophages have previously been shown to produce VEGF, and anti-VEGF therapy reduced melanoma growth to levels of non-CD34-engrafted MISTRG mice.[11,15]

In the blood, NSG-Quad mice had a much higher frequency of human mast cells (55-fold higher) and human $CD33^+CD66b^-CD14^-HLA\text{-}DR^-$ myeloid cells compared to MISTRG-6 mice and healthy human adults. A similar high frequency of $Fc\varepsilon R1\alpha^+CD117^+$ mast cells has been reported in NSGS mice[21,22] and may be due to the transgenic overexpression of human SCF, which is the ligand for CD117 (also called c-Kit or SCF receptor). However, NSG-Quad mice had a severely reduced frequency of human $CD4^+$ and $CD8^+$ T cells in the blood and spleen, which is in contrast to NSGS mice that strongly support human $CD4^+$ T cell development.[22] Yet, NSG-Quad, MISTRG-6, and NSG mice similarly supported $CD4^+$ and $CD8^+$ T cell infiltration into CRC xenografts, thus indicating successful engraftment and migration of human T cells in NSG-Quad mice. NSG-Quad and MISTRG-6 mice showed a similarly high infiltration of $CD33^+$ myeloid cells into SW480 tumors, whereas $CD33^+$ myeloid cells were less frequent in HCT116 tumors. This may be explained by SW480 being an MSS tumor that produces GM-CSF (ATCC.org), whereas HCT116 is an MSI tumor that produces immunosuppressive cytokines, such as transforming growth factor β1 (TGFβ1) and TGFβ2.

In summary, NSG-Quad mice supported the development of tissue-resident and tumor-infiltrating macrophages at levels almost comparable to those of MISTRG-6 mice. However, a subset of NSG-Quad mice displayed a monocyte hyperactivation syndrome that was previously also found in a subset of NSGS mice. In contrast to MISTRG-6 mice, NSG-Quad mice did not improve human NK cell development in the blood, and the frequency of human $CD4^+$ and $CD8^+$ T cells was reduced in the blood and spleen but not in the tumor of NSG-Quad mice.

MATERIALS AND METHODS

Humanized mice

NSG mice (NOD.Cg-$Prkdc^{scid}$ $Il2rg^{tm1Wjl}$/SzJ; JAX #005557), NSGS mice (also known as NSG-SGM3; NOD.Cg-$Prkdc^{scid}$ $Il2rg^{tm1Wjl}$ Tg (CMV-IL3,CSF2,KITLG)1Eav/MloySzJ; JAX #013062), and NSG-Quad mice (NOD.Cg-$Prkdc^{scid}$ $Il2rg^{tm1Wjl}$ Tg(CMV-IL3,CSF2, KITLG)1Eav Tg(CSF1)3Sz/J; JAX #028657) were purchased from the Jackson Laboratory (JAX). MISTRG-6 mice (C; 129S4-$Rag2^{tm1.1Flv}$ $Il2rg^{tm1.1Flv}$/J M-$CSF^{h/h}$ IL-3/GM-$CSF^{h/h}$ $SIRP\alpha^{h/m}$ $THPO^{h/h}$ IL-$6^{h/m}$) (VG5097/5090/5155/5089/5079/5078/790) were generated by Regeneron Pharmaceuticals in collaboration with the Richard A. Flavell lab at Yale University using VelociGene Technology.[11,13,23] The development and characterization of NSG,[24,25] NSGS,[22,26] NSG-Quad,[16,27,28] and MISTRG-6 mice[11,13,20,29–31] have been described, and two recent reviews highlighted the advantages and disadvantages of these strains.[7,8] Briefly, NSGS mice express human IL-3, GM-CSF, and SCF as homozygous transgenes, each with a human cytomegalovirus promoter/enhancer sequence. NSG-Quad mice are NSGS mice that additionally express the human M-CSF transgene, which appears to utilize an endogenous promoter.[16,27] MISTRG-6 mice harbor five human gene knock-ins on a Balb/c x 129 $Rag2^{-/-}$ $Il2rg^{-/-}$ background. The human genes are expressed by the endogenous mouse promoter, which allows tissue- and cell-specific expression of the human cytokines at physiological levels. For the generation of experimental $MIS^{h/m}TRG\text{-}6^{h/m}$ mice (homozygous for human M-CSF, IL-3/GM-CSF, and THPO; heterozygous for human SIRPα and IL-6), MITRG mice (homozygous for human M-CSF, IL-3/GM-CSF, and THPO) were crossed with MISTRG-6 mice (homozygous for human M-CSF, IL-3/GM-CSF, SIRPα, THPO, and IL-6). $SIRP\alpha^{h/m}$ heterozygous mice were used because $SIRP\alpha^{h/h}$ mice, which are mouse SIRPα knockout, show decreased human $CD34^+$ cell engraftment due to a defect of the bone marrow niche.[20,32] For the generation of experimental NSG-Quad mice, NSG-$Quad^{+/-}$ (heterozygous for human M-CSF) were crossed with NSG-$Quad^{+/-}$ mice, as recommended by the Jackson Laboratory due to fertility issues of NSG-$Quad^{+/+}$ mice. The fertility issues are caused by the human M-CSF transgene, since NSG M-$CSF^{+/+}$ mice have the same fertility issues. NSG-Quad mice heterozygous or homozygous for human M-CSF were used for our experiments, as indicated. All animal experiments have been approved by the Austrian Federal Ministry of Education, Science and Research (GZ 66.009/0408-V/3b/2018 and GZ 66.009/0409-V/3b/2018).

Human peripheral blood samples

Human peripheral blood samples were collected from systemically healthy volunteers (age 26–29 years, three women, one man).

Informed written consent was obtained from all study participants, and the study was approved by the ethics committee of the Medical University of Vienna (ECS #1874/2023).

Human-cord-blood-derived CD34+ cells

Human cord blood samples were collected from systemically healthy volunteers. Informed written consent was obtained from the mothers, and the study was approved by the ethics committee of the Medical University of Vienna (ECS #1692/2021). Human $CD34^+$ HSPCs were isolated from cord blood using an EasySep Human Cord Blood CD34 Positive Selection Kit II (StemCell Technologies, #17896). Briefly, $CD34^+$ cells were enriched by density gradient centrifugation using Lymphoprep (StemCell Technologies, #07861) followed by a positive magnetic selection with anti-human CD34 microbeads. The purity of the $CD34^+$ cell population was validated by flow cytometry. The $CD34^+$ cells were frozen in FBS containing 10% DMSO and stored in liquid nitrogen until use.

Generation of human immune system mice

Newborn NSG, NSGS, and NSG-Quad mice were sublethally irradiated with 100 cGy using a Yxlon irradiator. Thereafter, newborn NSG, NSGS, NSG-Quad, and MISTRG-6 mice were engrafted by intrahepatic injection of human-cord-blood-derived $CD34^+$ HSPCs. Intrahepatic injection of 1×10^5 $CD34^+$ cells for NSG and $5–6x10^4$ $CD34^+$ cells for NSGS, NSG-Quad, and MISTRG-6 in 20 μL of PBS was performed using a 22-gauge needle (Hamilton). We used engraftment protocols that have been optimized for individual mouse strains.[11,20,33] A high percentage of MISTRG-6 mice successfully engraft cord-blood-derived $CD34^+$ cells without the need for preconditioning, and in the absence of sublethal irradiation, anemia-related lethality is reduced. Accordingly, human $CD45^+$ cell engraftment levels were comparable between all four humanized mouse strains. Engraftment level and peripheral blood immune cell composition were determined 8 weeks post-engraftment by retroorbital bleeding and flow cytometric analysis. Mice with a percentage of human $CD45^+$ cells among total (mouse and human combined) $CD45^+$ cells of $\geq$10% were considered sufficiently engrafted and selected for further experimentation and analyses. In total, six independent experiments were performed, of which three received a pool of three different $CD34^+$ cell donors, respectively. The remaining three experiments comparing MISTRG-6 with NSG-Quad mice were engrafted each with one $CD34^+$ cell donor. Mice were maintained in a specific pathogen-free (SPF) environment at the Center for Biomedical Research of the Medical University of Vienna. Animals of both sexes were included in the experiments.

Blood and tissue sample preparation

To determine RBC counts, hemoglobin concentration, and hematocrit, blood was collected via cardiac puncture using an insulin syringe and 0.25/0.5 mL K_3EDTA MiniCollect tubes (Greiner Bio-One) and analyzed using a scil Vet abc hematology system (LabTechnologies). For immunophenotypic evaluation, blood was treated twice with ammonium-chloride-potassium (ACK) lysis buffer to eliminate RBCs prior to flow cytometric analysis. Intracardial perfusion with PBS was performed prior to the collection of liver and lung samples for immunohistochemistry. Tissues were stored in Histofix (Roth, P087.3) overnight and embedded in paraffin for immunohistochemistry. Spleen was dissociated and passed through a 100 μm nylon net filter (Merck, NY1H00010) to obtain a single-cell suspension in RPMI 1640 medium with 10% FBS and 1% Penicillin/Streptomycin (R10). Bone marrow of tibia and femur was collected by flushing the bones with PBS. After treatment of spleen and bone marrow with ACK lysis buffer, cells were counted using a Neubauer chamber and analyzed via flow cytometry.

Human tumor xenograft experiments

The human CRC cell lines SW480 (CCL-228; ATCC) and HCT116 (CCL-247; ATCC) were obtained from the Center for Cancer Research, and STR profiling was performed to authenticate the cell lines. SW480 is an MSS CRC with mutations in TP53, KRAS, C-MYC, MYB, FOS, and SIS oncogenes and expression of GM-CSF. HCT116 is an MSI CRC with mutations in KRAS and PIC3CA and expression of TGFβ1 and TGFβ2 (ATCC.org). Cells were incubated in R10 medium at 37°C and 5% CO_2. After reaching 80% confluence, the cells were used for subcutaneous injection. Human immune cell engraftment was analyzed at 7–8 weeks of age, and at week 9, 2×10^6 CRC cells were injected subcutaneously into the right flank of the mice, and caliper measurement was performed to assess tumor growth. Tumor volume was calculated using the following formula: $0.5 \times \text{length} \times \text{width}^2$. Tumors were removed and analyzed when reaching a tumor volume of approximately 1,000 mm^3 or when health issues (e.g., anemia or ulceration of the tumor) led to early termination of experiments. Upon removal, one-third of the tumor was stored in Histofix (Roth, P087.3) overnight and embedded in paraffin for immunohistochemistry. The remaining tumor sample was digested with collagenase type 4 (Worthington, LS004188, 5 mg/mL) at a ratio of 1:5 in R10 medium at 37°C for 45 min. Thereafter, the cell suspension was passed through a 70 μm cell strainer (Fisherbrand, 22363548).

Flow cytometry

Immunophenotypic evaluation of blood, spleen, and tumor was performed using a 10-color staining panel and a 4-laser LSRFortessa X-20 flow cytometer (BD Biosciences) or a 4-laser Gallios flow cytometer (Beckman Coulter). In-depth analysis of the human myeloid cell composition in the blood of NSG-Quad, MISTRG-6, and human adults was performed using an 18-color staining panel and a 5-laser Aurora full spectrum flow cytometer (Cytek Biosciences). Dead cells were excluded using 7-aminoactinomycin D (7-AAD; 420404), Zombie NIR (423105), or Ghost Dye 510 (13-0870-T100). Anti-human CD45 (hCD45, clone HI30, BV785, or BUV395) and anti-mouse CD45 (mCD45, clone 30-F11, BV421, or AF700) antibodies were used to determine human immune cell reconstitution (percentage of $hCD45^+$ cells of human + mouse $CD45^+$ cells). Human T, B, and NK cells were identified using antibodies recognizing CD3

(UCHT1, FITC), CD4 (SK3 or OKT4, AF700), CD8 (SK1, PE-Cy7), CD20 (2H7, APC/Fire750), CD56 (5.1H11, PE), and PD-1 (EH12.2H7, PE). Human myeloid cells were characterized using antibodies recognizing CD14 (M5E2, BV605 or 63D3, cFluor B548), CD16 (3G8, PerCP), CD33 (P67.6, APC or WM53, BV650), CD66b (6/40c, PE/Fire640), CD86 (BU63, FITC or BV421), CD117 (YB5.B8, BUV737), CD163 (GHI/61, BV785), FcεR1α (AER-37, BUV563), HLA-DR (L243, PerCP-Cy5.5), PD-L1 (MIH2, PE), SIGLEC10 (5G6, PE-Cy7), SIRPA (15–414, APC), TIM-3 (F38-2E2, BV421), and TREM2 (237920R, AF594). Human $CD34^+$ HSPC subpopulations in the bone marrow were identified using antibodies recognizing CD34 (561, APC-Cy7) and CD38 (S17015F, FITC) and a lineage cocktail to exclude red blood cells and mature cells. The lineage cocktail contained antibodies recognizing CD3 (SK7, PE), CD4 (A161A1, APC), CD33 (WM53, BV650), CD56 (HCD56, BV650), and TER-119 (TER-119, BV421). Mouse myeloid cells were characterized using antibodies recognizing CD11b (M1/70, FITC), CD11c (N418, APC/Fire750), F4/80 (BM8, PE), Ly6C (HK1.4, PE-Cy7), Ly6G (1A8, PacificBlue), and MHC class II antigen $I\text{-}A^d$ (AMS-32.1, APC). The fluorochrome-conjugated antibodies were obtained from Biolegend, BD Biosciences, Cytek Biosciences, Thermo Fisher Scientific, and R&D Systems. Data acquisition was performed using FACSDiva (BD Biosciences) or SpectroFlo software (Cytek Biosciences). Flow cytometry data were analyzed using FlowJo software (BD Biosciences).

Immunohistochemistry

FFPE samples were collected from liver, lung, and tumor of humanized mice. Tissue samples were cut into 4 μm sections and analyzed for CD68-expressing tissue-resident macrophages. We used the pan-monocyte/macrophage marker CD68, which has been shown to reliably detect tissue-resident and tumor-infiltrating monocytes/macrophages in FFPE samples.[34–36] Briefly, after deparaffinization of samples, antigen retrieval with Tris-EDTA buffer pH = 9 was performed. Endogenous peroxidase activity and unspecific antibody binding were blocked with 3% H_2O_2 (Merck, 107209) and blocking buffer containing 5% goat serum, 2% BSA, and 0.1% Triton in PBS, respectively. Anti-human CD68 antibody (Abcam, EPR20545) at a dilution of 1:4,000 (tumor) or 1:8,000 (liver, lung) was added to the tissue sections overnight and subsequently detected using an UltraVision LP Detection System (Epredia, TL-125-HL). For chromogenic reaction, 2% diaminobenzidine tetrahydrochloride (DAB, Dako Omnis, K3468) was used, and samples were counterstained with hematoxylin. Slides were scanned using a PANNORAMIC SCAN II digital slide scanner (3DHISTECH) and quantified using QuPath software.[37]

ELISA

For cytokine quantification, undiluted blood serum and bone marrow cell culture supernatants were collected and analyzed using commercially available AuthentiKine human M-CSF (Proteintech, KE00184), human GM-CSF (Thermo Fisher Scientific, 88–8337), and human IL-6 ELISA kits (Thermo Fisher Scientific, 88–7066). Bone marrow cells were collected as described previously[38] and plated in 96-well plates at 5×10^5 cells per well using RPMI 1640 medium supplemented with 10% fetal bovine serum and 1% Penicillin/Streptavidin. Bone marrow cells were stimulated with 100 ng/mL LPS (Sigma-Aldrich) overnight at 37°C and 5% CO_2. The supernatants were collected and stored for up to 1 week at −20°C until use. Secreted human cytokine levels were assayed by ELISA according to the manufacturer's protocol. Bone marrow supernatant samples were added in duplicates and incubated for 2 h at room temperature. The ELISA sensitivity was 1.1 pg/mL (human M-CSF), 6 pg/mL (human GM-CSF), and 2 pg/mL (human IL-6).

Statistical analysis

Data are presented as means with error bars indicating the standard error of the mean (SEM). The Shapiro-Wilk normality test was used to test for normal (Gaussian) distribution. Accordingly, one-way ANOVA with Tukey's multiple comparison test and Kruskal-Wallis with Dunn's correction test were used for comparing multiple groups with parametric and non-parametric data, respectively. Two-tailed, unpaired Student's t test and two-tailed, unpaired Mann-Whitney U test were used for comparing two groups with parametric and non-parametric data, respectively. Statistical analyses were performed using Prism 10 software (GraphPad).

DATA AVAILABILITY

All the data generated and analyzed in this study are included in the manuscript and/or the supplemental information. Raw data are available upon request from the corresponding author.

ACKNOWLEDGMENTS

We thank Regeneron Pharmaceuticals and the Richard A. Flavell lab at Yale University for generating and sharing the MISTRG-6 mice. We thank the staff of the histology, imaging, and cell culture facilities at the Center for Cancer Research (Medical University of Vienna) as well as the members of the Herndler-Brandstetter lab for their support and advice. Illustrations were created with BioRender.com. A.C. and V.K. were supported by an IPPTO PhD fellowship from the Austrian Science Fund (FWF). M.F. was supported by a DOC fellowship of the Austrian Academy of Sciences (No. 26398). D.H.-B. and C.B. were supported by the Austrian Science Fund (FWF): 10.55776/DOC59. D.H.-B. was supported by the Vienna Science and Technology Fund (WWTF) [10.47379/LS20042], the FWF (10.55776/P33340 and 10.55776/P36995), and the Fellinger Cancer Research Foundation.

AUTHOR CONTRIBUTIONS

A.C., V.K., C.B., and D.H.-B. designed the experiments. A.C., V.K., O.W., J.H., I.N., M.F., N.B., S.D., and C.S. performed the experiments. J.R. provided technical assistance. A.F. provided clinical samples. A.C., V.K., O.W., J.H., I.N., N.B., and D.H.-B. analyzed the data. A.C., V.K., C.B., and D.H.-B. interpreted the data. A.C. and D.H.-B. wrote the manuscript. D.H.-B. supervised the work. All authors discussed the data, commented on the manuscript, and approved the final version.

DECLARATION OF INTERESTS

The authors declare no competing financial interests.

SUPPLEMENTAL INFORMATION

Supplemental information can be found online at https://doi.org/10.1016/j.omtm.2025.101487.

REFERENCES

1. Qian, B.Z., and Pollard, J.W. (2010). Macrophage diversity enhances tumor progression and metastasis. Cell *141*, 39–51.
2. Zhang, Y., and Zhang, Z. (2020). The history and advances in cancer immunotherapy: understanding the characteristics of tumor-infiltrating immune cells and their therapeutic implications. Cell. Mol. Immunol. *17*, 807–821.
3. Ruffell, B., and Coussens, L.M. (2015). Macrophages and therapeutic resistance in cancer. Cancer Cell *27*, 462–472.
4. Bruni, D., Angell, H.K., and Galon, J. (2020). The immune contexture and Immunoscore in cancer prognosis and therapeutic efficacy. Nat. Rev. Cancer *20*, 662–680.
5. Ganesh, K., Stadler, Z.K., Cercek, A., Mendelsohn, R.B., Shia, J., Segal, N.H., and Diaz, L.A., Jr. (2019). Immunotherapy in colorectal cancer: rationale, challenges and potential. Nat. Rev. Gastroenterol. Hepatol. *16*, 361–375.
6. Andre, T., Shiu, K.K., Kim, T.W., Jensen, B.V., Jensen, L.H., Punt, C., Smith, D., Garcia-Carbonero, R., Benavides, M., Gibbs, P., et al. (2020). Pembrolizumab in Microsatellite-Instability-High Advanced Colorectal Cancer. N. Engl. J. Med. *383*, 2207–2218.
7. Chuprin, J., Buettner, H., Seedhom, M.O., Greiner, D.L., Keck, J.G., Ishikawa, F., Shultz, L.D., and Brehm, M.A. (2023). Humanized mouse models for immuno-oncology research. Nat. Rev. Clin. Oncol. *20*, 192–206.
8. Chen, A., Neuwirth, I., and Herndler-Brandstetter, D. (2023). Modeling the Tumor Microenvironment and Cancer Immunotherapy in Next-Generation Humanized Mice. Cancers (Basel) *15*, 2989.
9. Marin-Jimenez, J.A., Capasso, A., Lewis, M.S., Bagby, S.M., Hartman, S.J., Shulman, J., Navarro, N.M., Yu, H., Rivard, C.J., Wang, X., et al. (2021). Testing Cancer Immunotherapy in a Human Immune System Mouse Model: Correlating Treatment Responses to Human Chimerism, Therapeutic Variables and Immune Cell Phenotypes. Front. Immunol. *12*, 607282.
10. Rongvaux, A., Takizawa, H., Strowig, T., Willinger, T., Eynon, E.E., Flavell, R.A., and Manz, M.G. (2013). Human hemato-lymphoid system mice: current use and future potential for medicine. Annu. Rev. Immunol. *31*, 635–674.
11. Rongvaux, A., Willinger, T., Martinek, J., Strowig, T., Gearty, S.V., Teichmann, L.L., Saito, Y., Marches, F., Halene, S., Palucka, A.K., et al. (2014). Development and function of human innate immune cells in a humanized mouse model. Nat. Biotechnol. *32*, 364–372.
12. Ushach, I., and Zlotnik, A. (2016). Biological role of granulocyte macrophage colony-stimulating factor (GM-CSF) and macrophage colony-stimulating factor (M-CSF) on cells of the myeloid lineage. J. Leukoc. Biol. *100*, 481–489.
13. Yu, H., Borsotti, C., Schickel, J.N., Zhu, S., Strowig, T., Eynon, E.E., Frleta, D., Gurer, C., Murphy, A.J., Yancopoulos, G.D., et al. (2017). A novel humanized mouse model with significant improvement of class-switched, antigen-specific antibody production. Blood *129*, 959–969.
14. Voillet, V., Berger, T.R., McKenna, K.M., Paulson, K.G., Tan, W.H., Smythe, K.S., Hunter, D.S., Valente, W.J., Weaver, S., Campbell, J.S., et al. (2022). An In Vivo Model of Human Macrophages in Metastatic Melanoma. J. Immunol. *209*, 606–620.
15. Chiorazzi, M., Martinek, J., Krasnick, B., Zheng, Y., Robbins, K.J., Qu, R., Kaufmann, G., Skidmore, Z., Juric, M., Henze, L.A., et al. (2023). Autologous humanized PDX modeling for immuno-oncology recapitulates features of the human tumor microenvironment. J. Immunother. Cancer *11*, e006921.
16. Svoboda, D.S., Barrasa, M.I., Shu, J., Rietjens, R., Zhang, S., Mitalipova, M., Berube, P., Fu, D., Shultz, L.D., Bell, G.W., and Jaenisch, R. (2019). Human iPSC-derived microglia assume a primary microglia-like state after transplantation into the neonatal mouse brain. Proc. Natl. Acad. Sci. USA *116*, 25293–25303.
17. Shultz, L.D., Keck, J., Burzenski, L., Jangalwe, S., Vaidya, S., Greiner, D.L., and Brehm, M.A. (2019). Humanized mouse models of immunological diseases and precision medicine. Mamm. Genome *30*, 123–142.
18. Janke, L.J., Imai, D.M., Tillman, H., Doty, R., Hoenerhoff, M.J., Xu, J.J., Freeman, Z. T., Allen, P., Fowlkes, N.W., Iacobucci, I., et al. (2021). Development of Mast Cell and Eosinophil Hyperplasia and HLH/MAS-Like Disease in NSG-SGM3 Mice Receiving Human CD34+ Hematopoietic Stem Cells or Patient-Derived Leukemia Xenografts. Vet. Pathol. *58*, 181–204.
19. Tarrant, J.C., Binder, Z.A., Bugatti, M., Vermi, W., van den Oord, J., Ranieri, B., Assenmacher, C.A., Hoepp, N., O'Rourke, D.M., Shan, X., et al. (2021). Pathology of macrophage activation syndrome in humanized NSGS mice. Res. Vet. Sci. *134*, 137–146.
20. Herndler-Brandstetter, D., Shan, L., Yao, Y., Stecher, C., Plajer, V., Lietzenmayer, M., Strowig, T., de Zoete, M.R., Palm, N.W., Chen, J., et al. (2017). Humanized mouse model supports development, function, and tissue residency of human natural killer cells. Proc. Natl. Acad. Sci. USA *114*, E9626–E9634.
21. Sippel, T.R., Radtke, S., Olsen, T.M., Kiem, H.P., and Rongvaux, A. (2019). Human hematopoietic stem cell maintenance and myeloid cell development in next-generation humanized mouse models. Blood Adv. *3*, 268–274.
22. Billerbeck, E., Barry, W.T., Mu, K., Dorner, M., Rice, C.M., and Ploss, A. (2011). Development of human CD4+FoxP3+ regulatory T cells in human stem cell factor-granulocyte-macrophage colony-stimulating factor-and interleukin-3-expressing NOD-SCID IL2Rgamma(null) humanized mice. Blood *117*, 3076–3086.
23. Valenzuela, D.M., Murphy, A.J., Frendewey, D., Gale, N.W., Economides, A.N., Auerbach, W., Poueymirou, W.T., Adams, N.C., Rojas, J., Yasenchak, J., et al. (2003). High-throughput engineering of the mouse genome coupled with high-resolution expression analysis. Nat. Biotechnol. *21*, 652–659.
24. Ito, M., Hiramatsu, H., Kobayashi, K., Suzue, K., Kawahata, M., Hioki, K., Ueyama, Y., Koyanagi, Y., Sugamura, K., Tsuji, K., et al. (2002). NOD/SCID/gamma(c)(null) mouse: an excellent recipient mouse model for engraftment of human cells. Blood *100*, 3175–3182.
25. Shultz, L.D., Lyons, B.L., Burzenski, L.M., Gott, B., Chen, X., Chaleff, S., Kotb, M., Gillies, S.D., King, M., Mangada, J., et al. (2005). Human lymphoid and myeloid cell development in NOD/LtSz-scid IL2R gamma null mice engrafted with mobilized human hemopoietic stem cells. J. Immunol. *174*, 6477–6489.
26. Wunderlich, M., Chou, F.S., Link, K.A., Mizukawa, B., Perry, R.L., Carroll, M., and Mulloy, J.C. (2010). AML xenograft efficiency is significantly improved in NOD/SCID-IL2RG mice constitutively expressing human SCF, GM-CSF and IL-3. Leukemia *24*, 1785–1788.
27. Lee, J., Brehm, M.A., Greiner, D., Shultz, L.D., and Kornfeld, H. (2013). Engrafted human cells generate adaptive immune responses to Mycobacterium bovis BCG infection in humanized mice. BMC Immunol. *14*, 53.
28. Stocks, H., De Leeuw, E., Lambrecht, B.N., Vandekerckhove, L., van Loo, G., and Wullaert, A. (2024). Development of human innate immune responses in a humanized mouse model expressing four human myelopoiesis transgenes. Front. Immunol. *15*, 1419117.
29. Willinger, T., Rongvaux, A., Takizawa, H., Yancopoulos, G.D., Valenzuela, D.M., Murphy, A.J., Auerbach, W., Eynon, E.E., Stevens, S., Manz, M.G., and Flavell, R. A. (2011). Human IL-3/GM-CSF knock-in mice support human alveolar macrophage development and human immune responses in the lung. Proc. Natl. Acad. Sci. USA *108*, 2390–2395.
30. Rathinam, C., Poueymirou, W.T., Rojas, J., Murphy, A.J., Valenzuela, D.M., Yancopoulos, G.D., Rongvaux, A., Eynon, E.E., Manz, M.G., and Flavell, R.A. (2011). Efficient differentiation and function of human macrophages in humanized CSF-1 mice. Blood *118*, 3119–3128.
31. Rongvaux, A., Willinger, T., Takizawa, H., Rathinam, C., Auerbach, W., Murphy, A. J., Valenzuela, D.M., Yancopoulos, G.D., Eynon, E.E., Stevens, S., et al. (2011). Human thrombopoietin knockin mice efficiently support human hematopoiesis in vivo. Proc. Natl. Acad. Sci. USA *108*, 2378–2383.
32. Koskinen, C., Persson, E., Baldock, P., Stenberg, Å., Boström, I., Matozaki, T., Oldenborg, P.A., and Lundberg, P. (2013). Lack of CD47 impairs bone cell differentiation and results in an osteopenic phenotype in vivo due to impaired signal regulatory protein alpha (SIRPalpha) signaling. J. Biol. Chem. *288*, 29333–29344.
33. Sefik, E., Israelow, B., Mirza, H., Zhao, J., Qu, R., Kaffe, E., Song, E., Halene, S., Meffre, E., Kluger, Y., et al. (2022). A humanized mouse model of chronic COVID-19. Nat. Biotechnol. *40*, 906–920.
34. Wang, X., Yuwen, T.J., Zhong, Y., Li, Z.G., and Wang, X.Y. (2023). A new method for predicting the prognosis of colorectal cancer patients through a combination of multiple tumor-associated macrophage markers at the invasive front. Heliyon *9*, e13211.

35. Hegarty, L.M., Jones, G.R., and Bain, C.C. (2023). Macrophages in intestinal homeostasis and inflammatory bowel disease. Nat. Rev. Gastroenterol. Hepatol. *20*, 538–553.

36. Lavin, Y., Mortha, A., Rahman, A., and Merad, M. (2015). Regulation of macrophage development and function in peripheral tissues. Nat. Rev. Immunol. *15*, 731–744.

37. Bankhead, P., Loughrey, M.B., Fernández, J.A., Dombrowski, Y., McArt, D.G., Dunne, P.D., McQuaid, S., Gray, R.T., Murray, L.J., Coleman, H.G., et al. (2017). QuPath: Open source software for digital pathology image analysis. Sci. Rep. *7*, 16878.

38. Cordeiro Gomes, A., Hara, T., Lim, V.Y., Herndler-Brandstetter, D., Nevius, E., Sugiyama, T., Tani-Ichi, S., Schlenner, S., Richie, E., Rodewald, H.R., et al. (2016). Hematopoietic Stem Cell Niches Produce Lineage-Instructive Signals to Control Multipotent Progenitor Differentiation. Immunity *45*, 1219–1231.

OMTM, Volume *33*

Supplemental information

Comparison of NSG-Quad and MISTRG-6 humanized mice for modeling circulating and tumor-infiltrating human myeloid cells

Anna Chen, Viktoria Knöbl, Oliver Walzer, Jana Hauser, Ines Neuwirth, Magdalena Frank, Nina Braun, Semina Duvnjak, Johannes Reisecker, Carmen Stecher, Alex Farr, Christine Brostjan, and Dietmar Herndler-Brandstetter

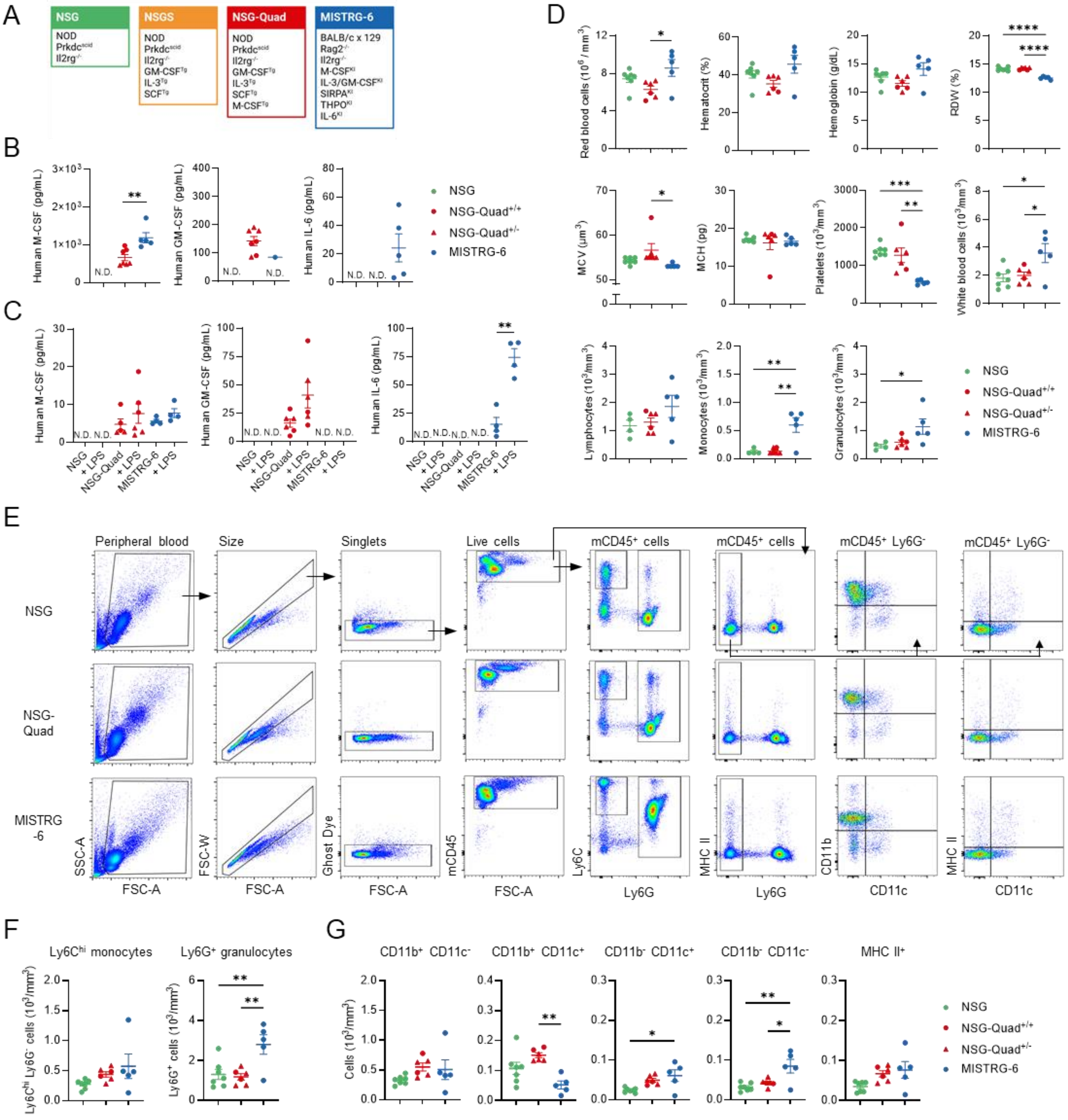

Figure S1: Characterization of NSG, NSG-Quad and MISTRG-6 mice (related to Figure 1). (**A**) Schematic illustration of the genetic background of NSG, NSGS (NSG-SGM3), NSG-Quad and MISTRG-6 mice. (**B**) Quantification of human M-CSF, GM-CSF and IL-6 protein in the plasma of 16-20 week old female NSG (n=5), NSG-Quad (dots, M-CSF$^{+/+}$ n=3; triangles, M-CSF$^{+/-}$ n=4) and MISTRG-6 mice (n=5). (**C**) Quantification of human M-CSF, GM-CSF and IL-6 protein in the supernatant of unstimulated and LPS-stimulated bone marrow cells from 16-20 week old female NSG (n=3), NSG-Quad (n=6) and MISTRG-6 mice (n=4). Triangle symbols

represent human M-CSF$^{+/-}$ mice. (**D**) Analysis of mouse red blood cells, hematocrit, hemoglobin, RDW, MCV, MCH, platelets, as well as mouse white blood cells, lymphocytes, monocytes and granulocytes in the blood of 16-20 week old female NSG (n=7), NSG-Quad (n=6) and MISTRG-6 mice (n=5). Triangle symbols represent human M-CSF$^{+/-}$ mice. (**E**) Representative flow cytometry plots showing the gating strategy for identifying mouse immune cell subsets in the blood of NSG, NSG-Quad and MISTRG-6 mice. (**F**) Absolute numbers of mouse Ly6C^{hi} Ly6G^{-} monocytes and Ly6G^{+} granulocytes in the blood of 16-20 week old female NSG (n=7), NSG-Quad (n=6) and MISTRG-6 mice (n=5). (**G**) Absolute numbers of mouse myeloid cell subsets in the blood of 16-20 week old female NSG (n=7), NSG-Quad (n=6) and MISTRG-6 mice (n=5). Data are shown as mean ± SEM. *P* values were calculated using one-way ANOVA with Tukey's multiple comparison test. * $P < 0.05$, ** $P < 0.01$, *** $P < 0.001$ and **** $P < 0.0001$. Abbreviations: CSF, colony-stimulating factor; GM-CSF, granulocyte-macrophage colony-stimulating factor; IL2rg, interleukin 2 receptor subunit gamma; KI, knock-in; MCH, mean corpuscular hemoglobin; M-CSF, macrophage colony-stimulating factor; MCV, mean corpuscular volume; N.D., not detected; NOD, non-obese diabetic; Prkdc, protein kinase DNA-activated catalytic subunit; Rag2, recombination activating gene 2; RDW, red blood cell distribution width; SCF, stem cell factor; scid, severe combined immunodeficiency; SIRPA, signal-regulatory protein alpha; Tg, transgene; THPO, thrombopoietin.

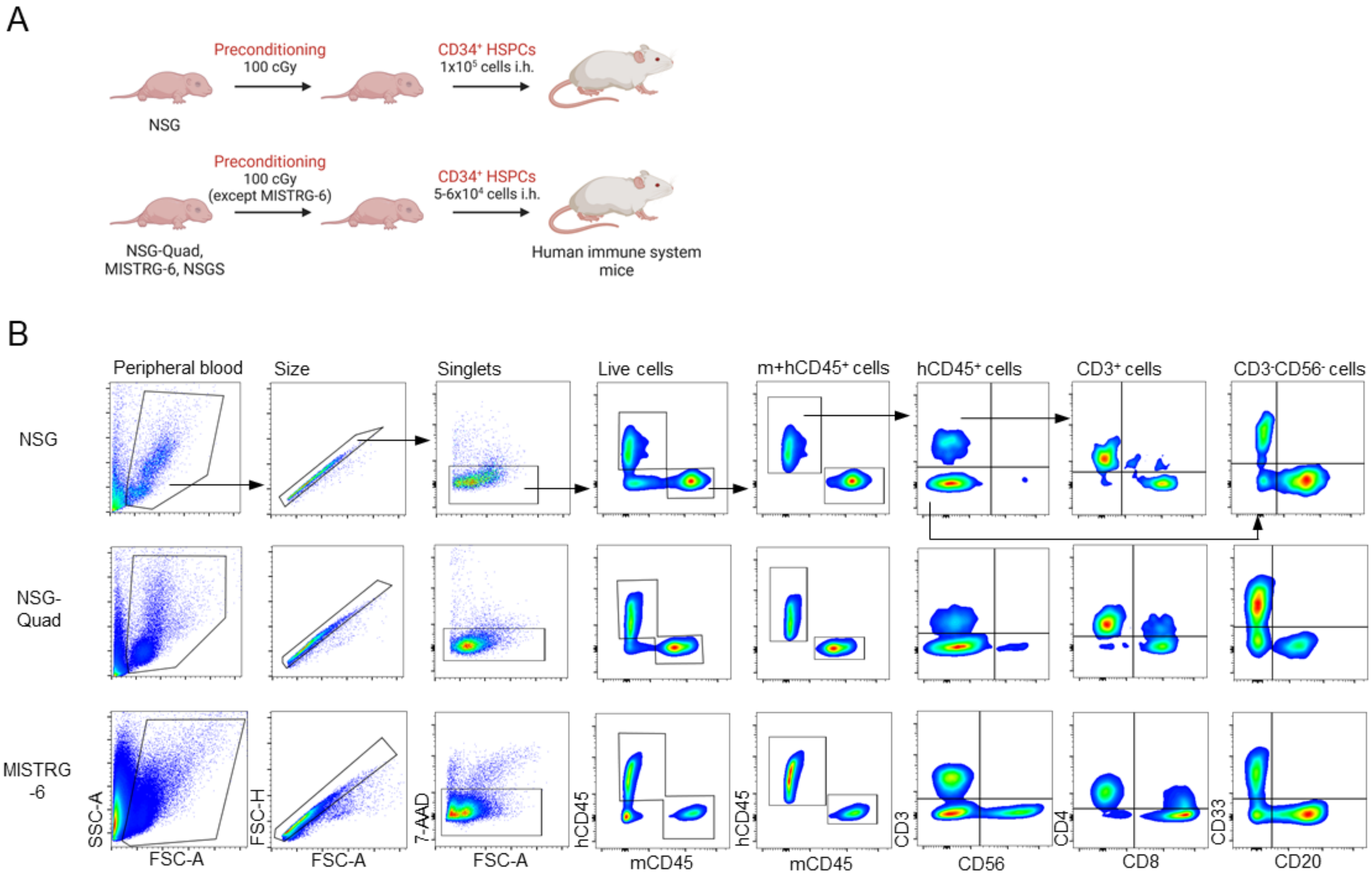

Figure S2: Human hematopoietic cell engraftment scheme and identification of human immune cell populations in NSG, NSG-Quad and MISTRG-6 mice (related to Figure 1). (**A**) Schematic illustration of the engraftment protocol with human cord blood-derived $CD34^{+}$ HSPCs into NSG, NSGS, NSG-Quad and MISTRG-6 mice. (**B**) Representative flow cytometry plots showing the gating strategy for identifying human immune cell subsets in the blood of NSG, NSG-Quad and MISTRG-6 mice. Abbreviations: cGy, centi Gray; HSPC, hematopoietic stem and progenitor cells; i.h., intrahepatic.

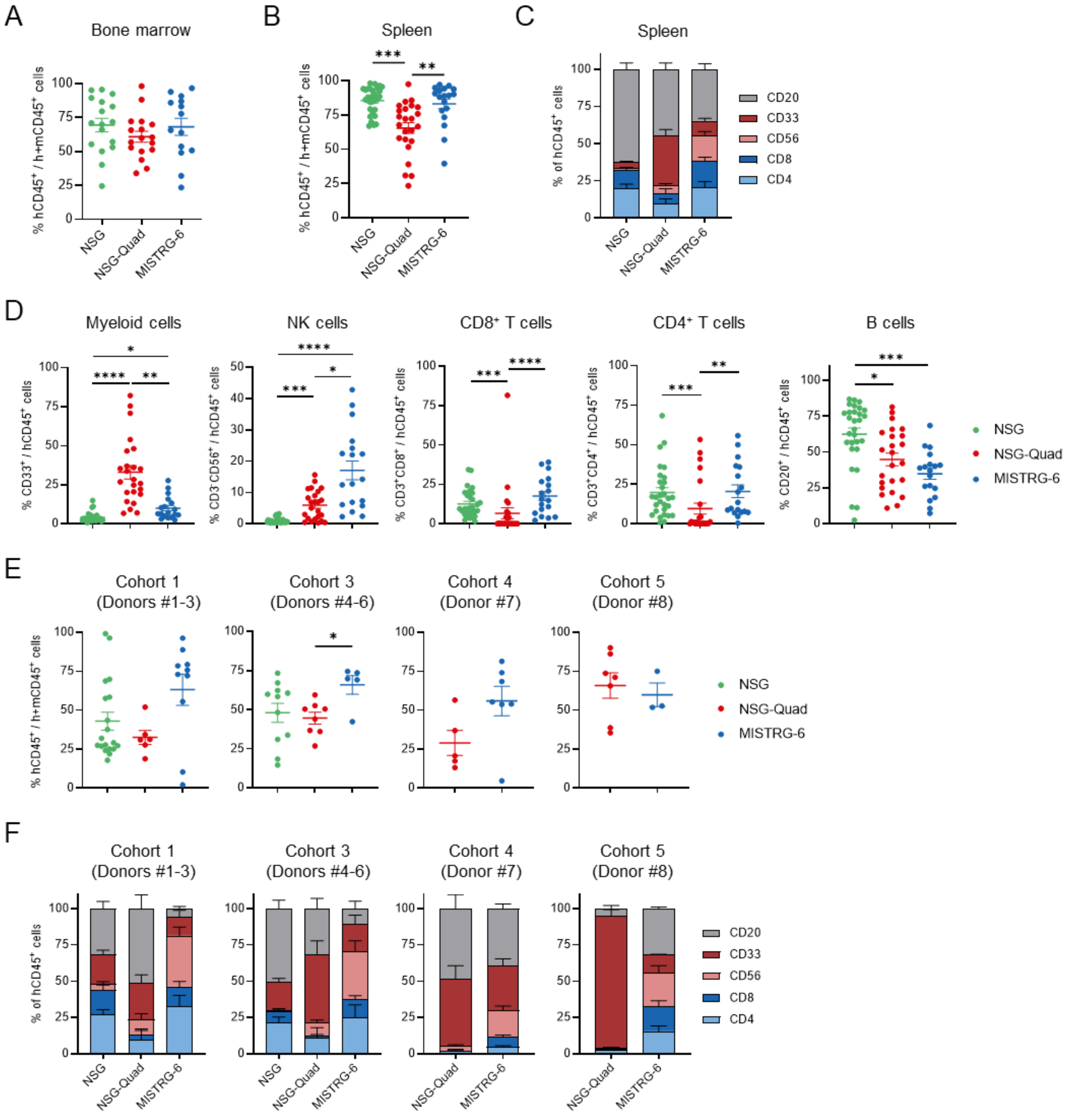

Figure S3: Human hematopoietic cell engraftment in the bone marrow, spleen and blood of NSG, NSG-Quad and MISTRG-6 mice (related to Figure 1). (**A**) Percentage of human CD45$^+$ (hCD45$^+$) cells of total CD45$^+$ cells (mouse and human) in the bone marrow of NSG (n=17), NSG-Quad (n=17) and MISTRG-6 mice (n=14) 10-15 weeks post engraftment with human cord blood-derived CD34$^+$ cells. (**B**) Percentage of human CD45$^+$ (hCD45$^+$) cells of total CD45$^+$ cells (mouse and human) in the spleen of NSG (n=29), NSG-Quad (n=24) and MISTRG-6 mice (n=18) 10-15 weeks post engraftment with human cord blood-derived CD34$^+$ cells. (**C**) Human immune cell composition in the spleen of NSG (n=29), NSG-Quad (n=24) and MISTRG-6 mice (n=18) 10-15 weeks post engraftment. (**D**) Percentage of human immune cell subsets in the spleen of

NSG, NSG-Quad and MISTRG-6 mice 10-15 weeks post engraftment (data from C). B cells ($CD3^-CD56^-CD33^-CD20^+$), myeloid cells ($CD3^-CD56^-CD20^-CD33^+$), NK cells ($CD3^-CD56^+$), $CD8^+$ T cells ($CD3^+CD4^-CD8^+$) and $CD4^+$ T cells ($CD3^+CD8^-CD4^+$) (data from C). (**E**) Percentage of human $CD45^+$ ($hCD45^+$) cells of total $CD45^+$ cells in the blood of NSG (n=11-19), NSG-Quad (n=5-8) and MISTRG-6 mice (n=3-10) 10-15 weeks post engraftment split by $CD34^+$ cell donors. (**F**) Human immune cell composition in the blood of NSG, NSG-Quad, and MISTRG-6 mice 10-15 weeks post engraftment split by $CD34^+$ cell donors (data from E). Data are shown as mean ± SEM. *P* values were calculated using Kruskal-Wallis with Dunn's correction test (A, B, D, E) and two-tailed, unpaired Student's *t*-test (E). * $P < 0.05$, ** $P < 0.01$, *** $P < 0.001$ and **** $P < 0.0001$.

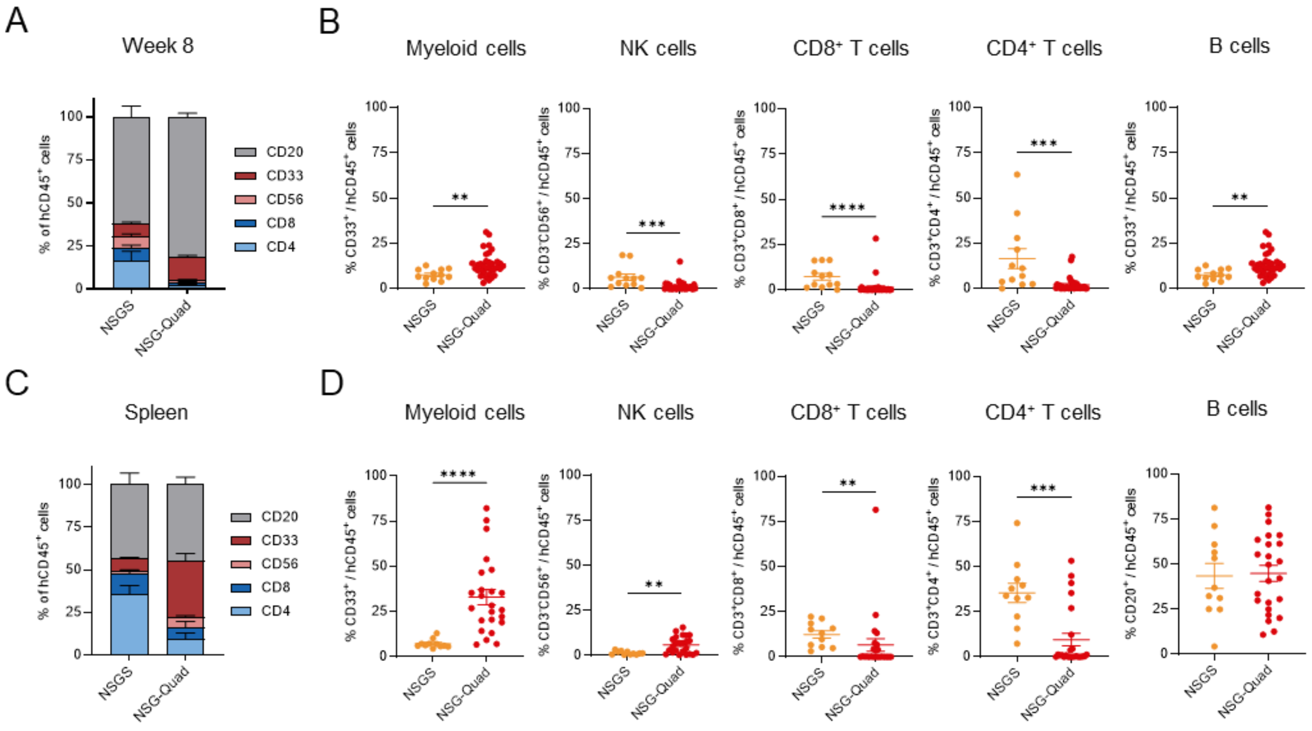

Figure S4: Human immune cell composition in NSGS and NSG-Quad humanized mice (related to Figure 2). (**A**) Human immune cell composition in the blood of NSGS (n=12) and NSG-Quad mice (n=34) 8 weeks post engraftment. (**B**) Percentage of human immune cell subsets in the blood of NSGS and NSG-Quad mice 8 weeks post engraftment (data from A). (**C**) Human immune cell composition in the spleen of NSGS (n=11) and NSG-Quad mice (n=24) 10-15 weeks post engraftment. (**D**) Percentage of human immune cell subsets in the spleen of NSGS and NSG-Quad mice 10-15 weeks post engraftment (data from C). Data are shown as mean ± SEM. *P* values were calculated using two-tailed, unpaired Mann-Whitney U test. ** $P < 0.01$, *** $P < 0.001$ and **** $P < 0.0001$.

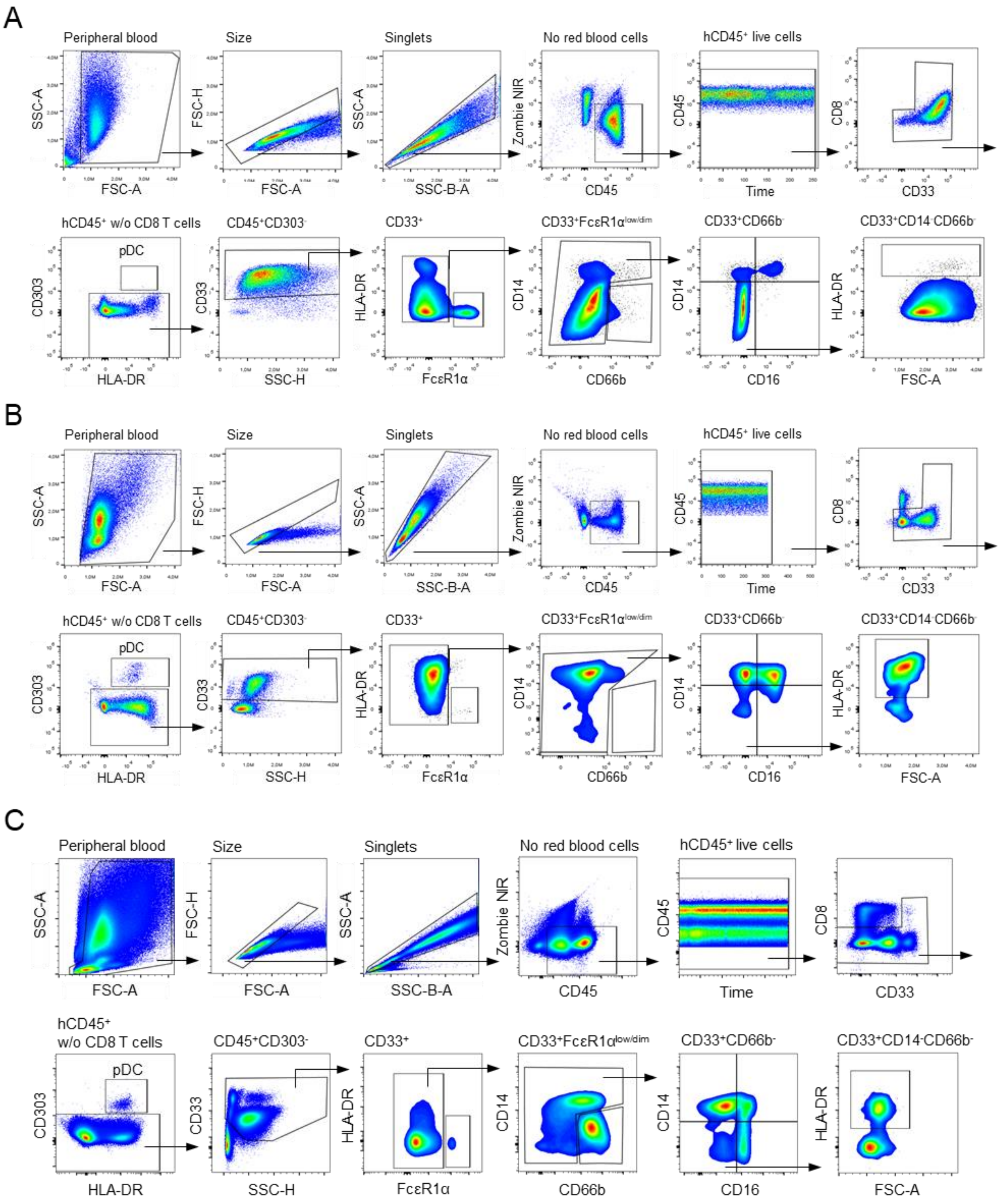

Figure S5: Gating strategy of human myeloid cell subpopulations in NSG-Quad, MISTRG-6 and healthy human adults (related to Figure 3). (**A**) Representative flow cytometry plots showing the gating strategy of human myeloid cell subpopulations in the blood of NSG-Quad mice. (**B**) Representative flow cytometry plots showing the gating strategy of human myeloid cell subpopulations in the blood of MISTRG-6 mice. (**C**) Representative flow cytometry plots showing the gating strategy of human myeloid cell subpopulations in the blood of healthy human adults.

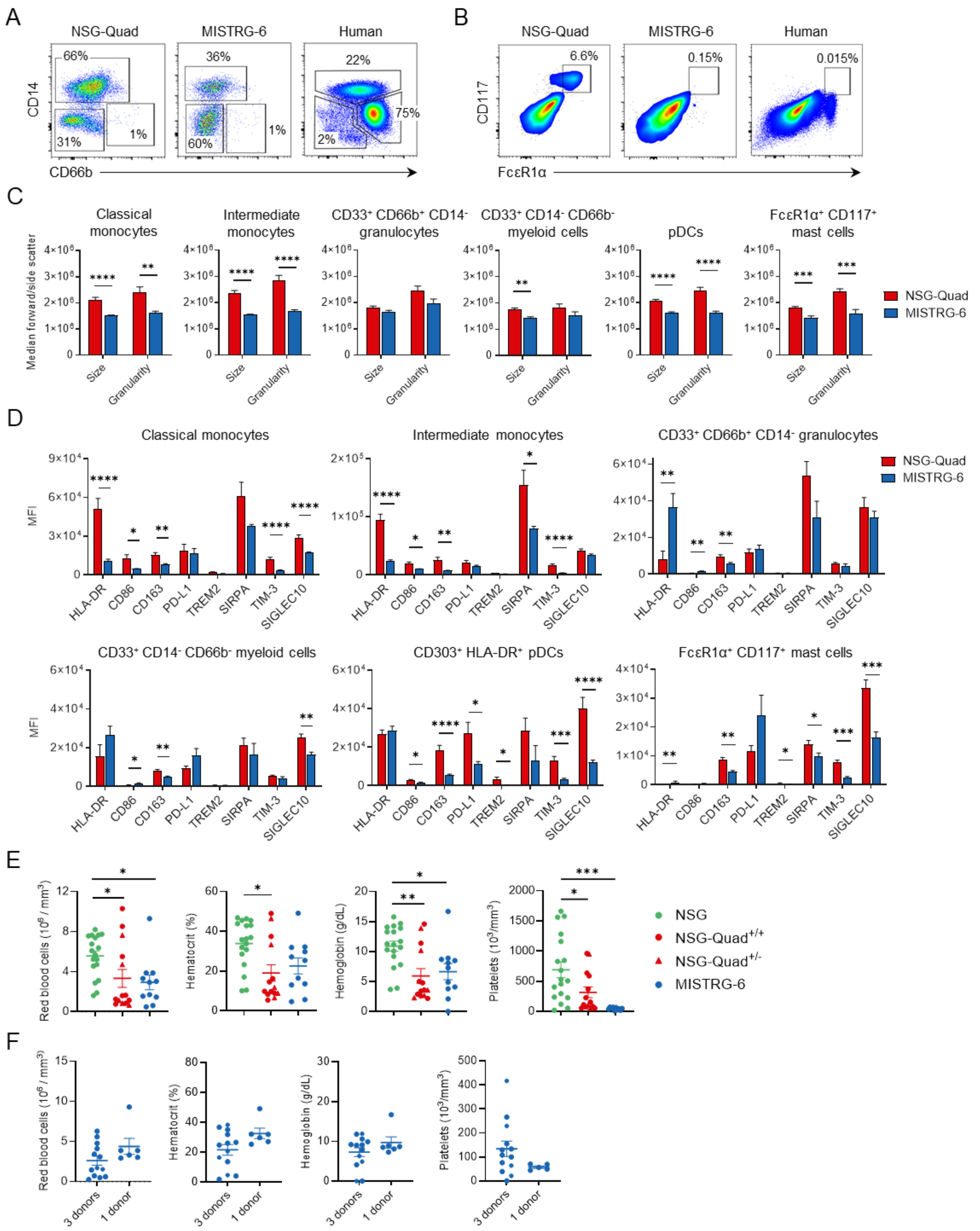

Figure S6: Expression pattern of key molecules in human myeloid cell subpopulations in NSG-Quad

and MISTRG-6 humanized mice (related to Figure 3). (**A**) Representative flow cytometry plots of $CD14^+$ monocytes, $CD66b^+$ granulocytes and $CD33^+CD14^-CD66b^-$ myeloid cells in the blood of NSG-Quad, MISTRG-6 and a healthy human adult. (**B**) Representative flow cytometry plots of $Fc\varepsilon R1\alpha^+CD117^+$ mast cells in NSG-Quad, MISTRG-6 and a healthy human adult. (**C**) Median forward scatter (cell size) and median side scatter (granularity) of six human myeloid cell populations in the blood of NSG-Quad (n=18) and MISTRG-6 mice (n=16) at 10-15 weeks post engraftment with human cord blood-derived $CD34^+$ cells. (**D**) Median fluorescence intensity (MFI) of eight key myeloid molecules on six human myeloid cell populations in the blood of NSG-Quad (n=18; mast cells n=15) and MISTRG-6 mice (n=16; mast cells n=12) at 10-15 weeks post engraftment with human cord blood-derived $CD34^+$ cells. (**E**) Analysis of mouse red blood cells, hematocrit, hemoglobin and platelets in the blood of hCD34-engrafted NSG (n=18), NSG-Quad (n=14) and MISTRG-6 mice (n=11). Triangle symbols represent M-$CSF^{+/-}$ mice. (**F**) Analysis of mouse red blood cells, hematocrit, hemoglobin and platelets in the blood of hCD34-engrafted MISTRG-6 mice (n=13 (3 donors); n=6 (1 donor)) split by $CD34^+$ cell donors. Data are shown as mean ± SEM. *P* values were calculated using two-tailed, unpaired Mann-Whitney U test (C, D, F) and Kruskal-Wallis with Dunn's correction test (E). * $P < 0.05$, ** $P < 0.01$, *** $P < 0.001$ and **** $P < 0.0001$.

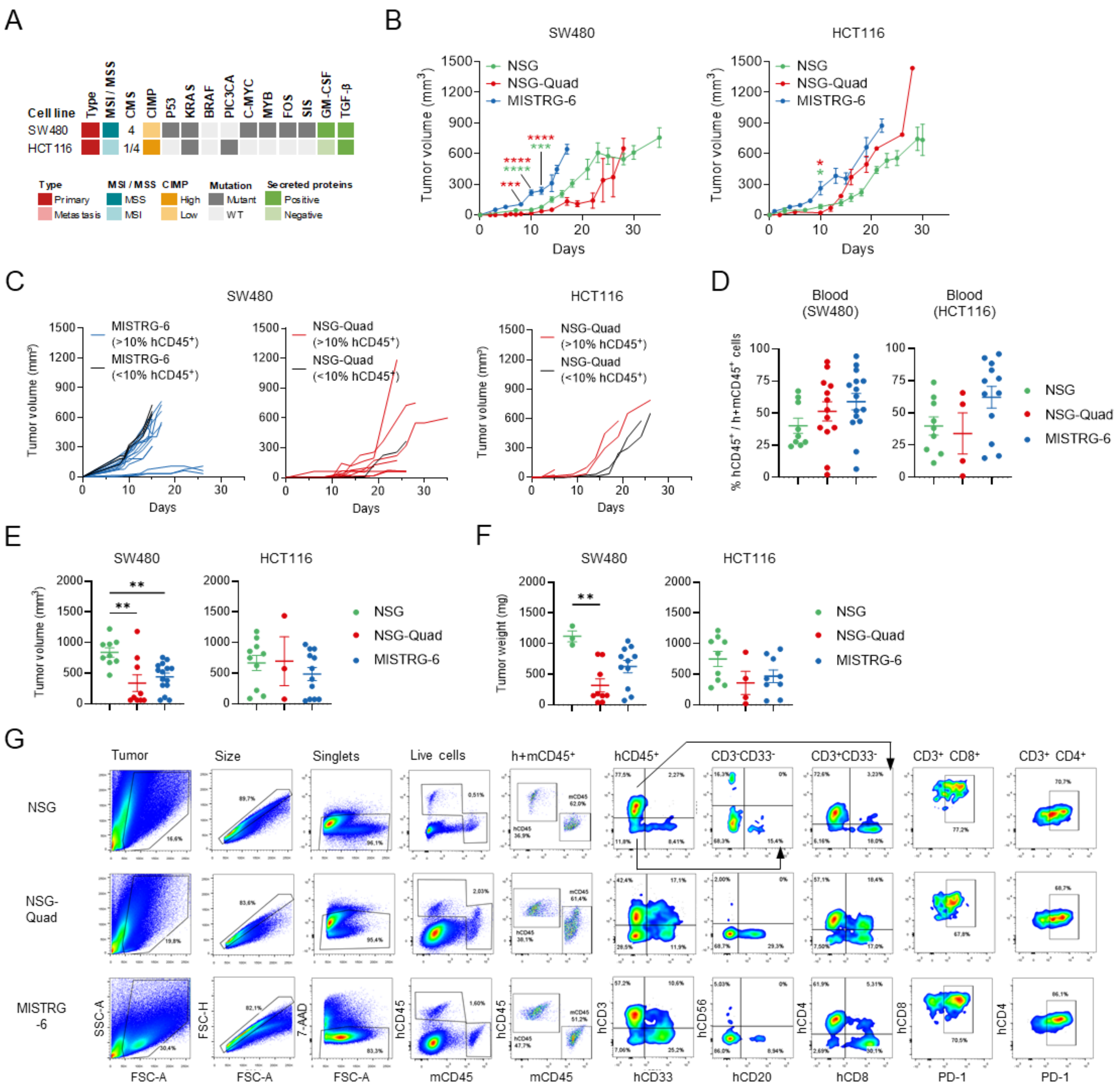

Figure S7: Human immune composition in the blood and tumor of NSG, NSG-Quad and MISTRG-6 humanized mice (related to Figure 5). (**A**) Characteristics of SW480 and HCT116 human CRC cell lines. (**B**) Average tumor growth curves in hCD34-engrafted NSG (n=9), NSG-Quad (n=12) and MISTRG-6 mice (n=15) engrafted with SW480 CRC cells and in NSG (n=10), NSG-Quad (n=4) and MISTRG-6 mice (n=14) engrafted with HCT116 CRC cells. A red asterisk indicates a significant difference between MISTRG-6 and NSG-Quad mice. A green asterisk indicates a significant difference between MISTRG-6 and NSG mice. (**C**) SW480 CRC growth curves in NSG-Quad and MISTRG-6 mice with high (>10%; MISTRG-6: mean=58.97%; NSG-Quad: mean=51.45%) and low (<10%; MISTRG-6: mean=5.89%; NSG-Quad: mean=5.78%) hCD45^{+} cell reconstitution in the blood (left two plots). HCT116 CRC growth curves in NSG-Quad mice with high

(>10%; mean=33.95%) and low (<10%; mean=7.08%) hCD45$^+$ cell reconstitution in the blood (right plot). (**D**) Percentage of hCD45$^+$ cells of total CD45$^+$ cells (mouse and human) in the blood of NSG (n=9 (SW480); n=9 (HCT116)), NSG-Quad (n=13 (SW480); n=4 (HCT116)) and MISTRG-6 humanized mice (n=15 (SW480); n=12 (HCT116)) at 10-15 weeks post engraftment with human cord blood-derived CD34$^+$ cells (end of experiment). (**E**) Tumor volume at the time of analysis (end of experiment) in NSG, NSG-Quad and MISTRG-6 mice. (**F**) Tumor weight at the time of analysis (end of experiment) in NSG, NSG-Quad and MISTRG-6 mice. (**G**) Representative flow cytometry plots showing the gating strategy for identifying human immune cell subsets in the tumor of NSG, NSG-Quad and MISTRG-6 mice. Data are shown as mean ± SEM. *P* values were calculated using one-way ANOVA with Tukey's multiple comparison test. * $P < 0.05$, ** $P < 0.01$, *** $P < 0.001$ and **** $P < 0.0001$. Abbreviations: CIMP, CpG island methylator phenotype; CMS, consensus molecular subtype; MSI, microsatellite instability; MSS, microsatellite stability; WT, wild-type.

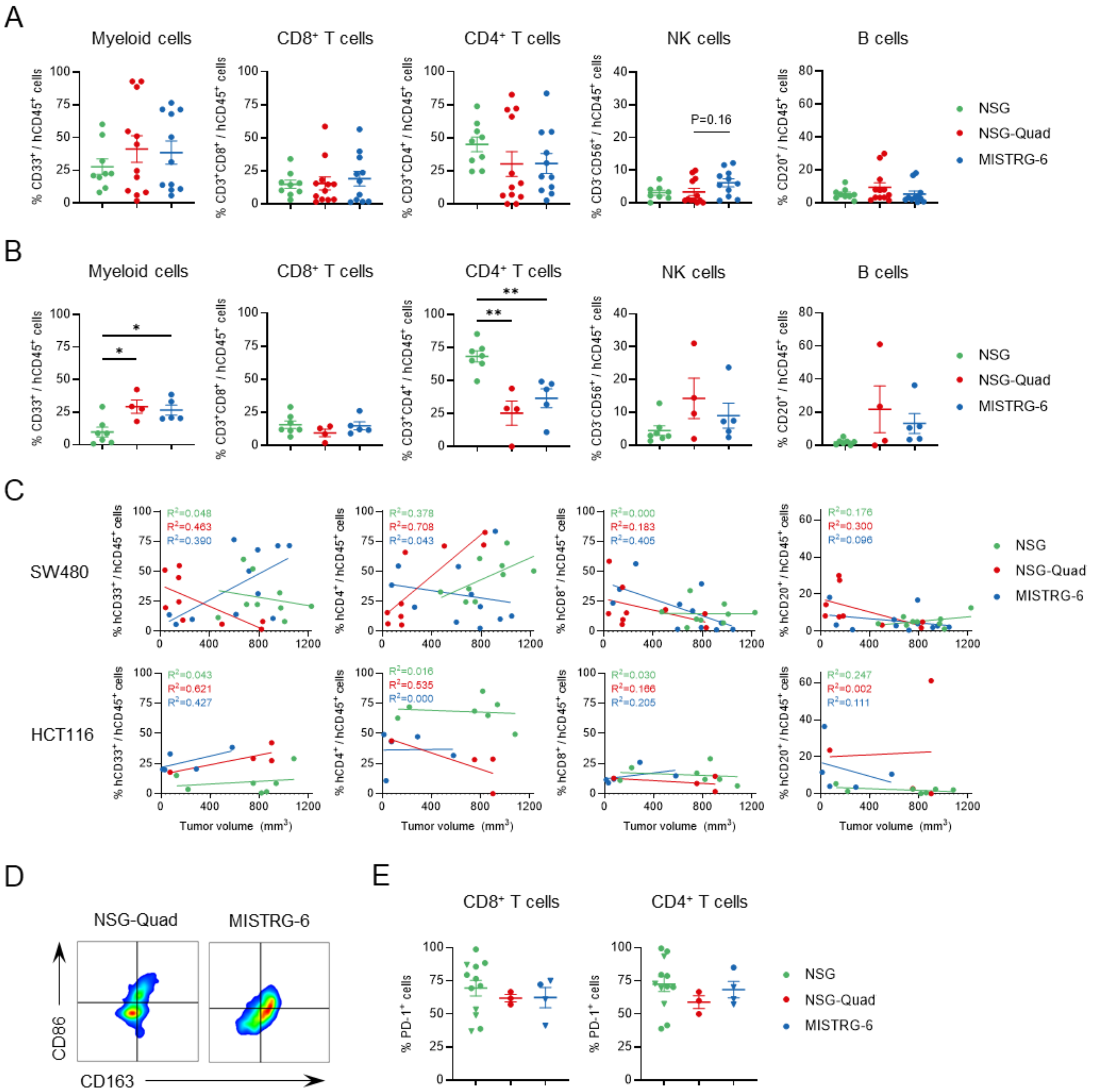

Figure S8: Human immune cell subsets in the tumor of NSG, NSG-Quad and MISTRG-6 humanized mice (related to Figure 5). (**A**) Percentage of human immune cell subsets in the tumor (SW480) of NSG (n=9), NSG-Quad (n=13) and MISTRG-6 humanized mice (n=11). (**B**) Percentage of human immune cell subsets in the tumor (HCT116) of NSG (n=9), NSG-Quad (n=4) and MISTRG-6 humanized mice (n=5). (**C**) Correlation of human immune cell subset frequencies with tumor volume in SW480 and HCT116 CRC xenografts of NSG, NSG-Quad and MISTRG-6 humanized mice. (**D**) Representative flow cytometry plots showing cells expressing CD86 and CD163 in human CD14^{+} monocytes in SW480 CRC xenografts of NSG-Quad and MISTRG-6 mice. (**E**) Frequency of PD-1-expressing CD8+ T cells and CD4+ T cells in SW480 and HCT116 CRC xenografts of NSG (n=12), NSG-Quad (n=3) and MISTRG-6 humanized mice (n=4). Dots

indicate mice with HCT116 tumors and triangles indicate mice with SW480 tumors. Data are shown as mean ± SEM. *P* values were calculated using one-way ANOVA with Tukey's multiple comparison test. Simple linear regression was performed and R^2 values calculated (C). * $P < 0.05$ and ** $P < 0.01$.

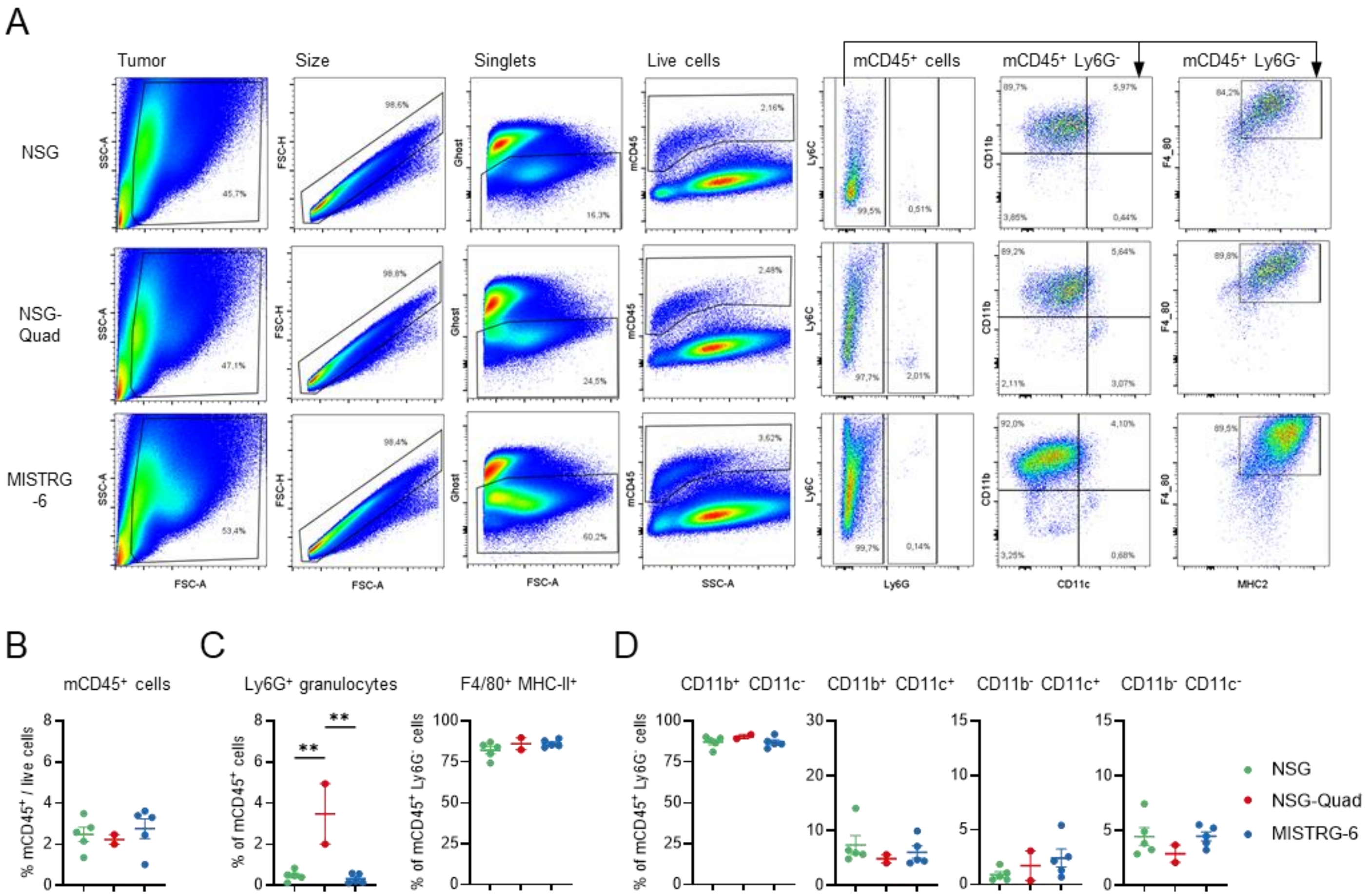

Figure S9: Mouse immune cell subsets in the tumor of NSG, NSG-Quad and MISTRG-6 humanized mice (related to Figure 5). (**A**) Representative flow cytometry plots showing the gating strategy for identifying mouse immune cell subsets in the tumor of NSG, NSG-Quad and MISTRG-6 mice. (**B**) Frequency of mCD45$^+$ cells in tumors of NSG, NSG-Quad and MISTRG-6 humanized mice engrafted with human SW480 CRC cells. (**C**) Frequency of mouse Ly6G$^+$ granulocytes and F4/80$^+$ MHC-II$^+$ macrophages in tumors of NSG, NSG-Quad and MISTRG-6 humanized mice engrafted with human SW480 CRC cells. (**D**) Frequency of four mouse myeloid cell subsets in tumors of NSG, NSG-Quad and MISTRG-6 humanized mice engrafted with human SW480 CRC cells. Data are shown as mean ± SEM. *P* values were calculated using one-way ANOVA with Tukey's multiple comparison test. ** $P < 0.01$.
